# Supplementary material for: Effects of repetitive transcranial magnetic stimulation over the contralesional dorsal premotor cortex on upper limb function in severe ischaemic stroke: study protocol for a randomised controlled trial
Source: BMJ Open. 2023 Dec 8;13(12):e074037. doi: 10.1136/bmjopen-2023-074037 (PMC10729250; doi:10.1136/bmjopen-2023-074037)
Supplement: Author's manuscript [file bmjopen-2023-074037.draft_revisions.pdf]

BMJ Open is committed to open peer review. As part of this commitment we make the peer review history of every article we publish publicly available.

When an article is published we post the peer reviewers' comments and the authors' responses online. We also post the versions of the paper that were used during peer review. These are the versions that the peer review comments apply to.

The versions of the paper that follow are the versions that were submitted during the peer review process. They are not the versions of record or the final published versions. They should not be cited or distributed as the published version of this manuscript.

BMJ Open is an open access journal and the full, final, typeset and author-corrected version of record of the manuscript is available on our site with no access controls, subscription charges or pay-per-view fees (http://bmjopen.bmj.com).

If you have any questions on BMJ Open's open peer review process please email info.bmjopen@bmj.com

BMJ Open

Effects of repetitive transcranial magnetic stimulation over the contralesional dorsal premotor cortex on upper limb function in severe stroke: study protocol for a randomized controlled trial

Journal:	*BMJ Open*
Manuscript ID	bmjopen-2023-074037
Article Type:	Protocol
Date Submitted by the Author:	29-Mar-2023
Complete List of Authors:	Dai, Wenjun; The First Affiliated Hospital of Nanjing Medical University, Rehabilitation Medicine Center<br>Liu, Canhuan; Nanjing Medical University, School of Rehabilitation Medicine<br>Yang, Xi; Nanjing Medical University, School of Rehabilitation Medicine<br>Ding, Hongyuan; The First Affiliated Hospital of Nanjing Medical University, Department of Radiology<br>Guo, Chuan; The First Affiliated Hospital of Nanjing Medical University, Rehabilitation Medicine Center<br>Zhu, Yi; The First Affiliated Hospital of Nanjing Medical Unviersity, Rehabilitation Medicine Center<br>Dong, Manyu; Nanjing Medical University, School of Rehabilitation Medicine<br>Qian, Yilun; Nanjing Medical University, School of Rehabilitation Medicine<br>Fang, Lu; The First Affiliated Hospital of Nanjing Medical University, Rehabilitation Medicine Center<br>Wang, Tong; The First Affiliated Hospital of Nanjing Medical University, Rehabilitation Medicine Center<br>Shen, Ying; The First Affiliated Hospital of Nanjing Medical University, Rehabilitation Medicine Center
Keywords:	Stroke < NEUROLOGY, REHABILITATION MEDICINE, Clinical trials < THERAPEUTICS

SCHOLARONE™
Manuscripts

Effects of repetitive transcranial magnetic stimulation over the contralesional dorsal premotor cortex on upper limb function in severe stroke: study protocol for a randomized controlled trial

Wenjun Dai[1#], Canhuan Liu[2#], Xi Yang[2#], Hongyuan Ding[3#], Chuan Guo[1], Yi Zhu[1], Manyu Dong[2], Yilun Qian[2], Lu Fang[1], Tong Wang[1*], Ying Shen[1*]

[1] Rehabilitation Medicine Center, The First Affiliated Hospital of Nanjing Medical University, Nanjing, China

[2] School of Rehabilitation Medicine, Nanjing Medical University, Nanjing, China

[3] Department of Radiology, The First Affiliated Hospital of Nanjing Medical University, Nanjing, China

[#]These authors have contributed equally to this work and share first authorship

*** Correspondence:**
Ying Shen
shenying@njmu.edu.cn
Tong Wang
wangtong60621@163.com

Abstract

Introduction: Repetitive transcranial magnetic stimulation (rTMS) is an evidence-based treatment widely recommended to promote hand motor recovery after stroke. However, the therapeutic efficacy of rTMS over the motor cortex in stroke patients is currently restricted and heterogeneous. This study aimed to determine whether excitatory rTMS over the contralesional dorsal premotor cortex (cPMd) facilitates the functional recovery of the upper limbs during the post-acute stage of severe stroke.

Methods and Analysis: This study will be conducted as a single-blind, controlled, randomized study, in which 44 patients with poststroke hemiplegia with a course of disease ranging from 1 week to 3 months and Fugl-Meyer upper limb score ≤22 will be enrolled. The study participants will be randomly assigned to groups A (n=22) and B (n=22). The two groups are based on routine rehabilitation training and drug treatment; group A will be treated with low-frequency (1 Hz) rTMS over the contralesional primary motor cortex (cM1), and group B will be treated with high-frequency rTMS (10 Hz) over cPMd. For 2 weeks, rTMS will be administered once a day, 5 days a week. The primary outcome is the Fugl–Meyer assessment of the upper limb. The secondary outcomes include the arm sub-score of the motricity index (AMI), Hong Kong edition of Functional Test for the Hemiplegic Upper Extremity (FTHUE-HK), Modified Barthel Index (MBI), and Modified Ashworth Scale (MAS) score of the paralyzed pectoralis major (PM) and biceps brachii (BI). Furthermore, data of diffusion tensor imaging (DTI) and functional magnetic resonance imaging (fMRI) will be collected. These outcomes will be assessed before and after the completion of the intervention.

Ethics and dissemination This study has been approved by the Ethics Committee of First Affiliated Hospital of Nanjing Medical University. The findings of this study will be spread through networks

For peer review only

of scientists, professionals, and the general public as well as peer-reviewed scientific papers and presentations at pertinent conferences.

Clinical Trial registration number ChiCTR2000038049

Strengths and limitations of this study

1. This is a single-blinded, controlled, randomized clinical study that probes the efficacy of high-frequency rTMS over the cPMd for restoring upper-limb function in severe stroke.
2. The study will recommend a more targeted rTMS approach to improve upper extremity movement after a severe stroke.
3. Multiple outcome measures, including clinical function and functional magnetic resonance imaging will be helpful to explore the neural mechanisms of TMS intervention.

1 Introduction

Stroke has emerged as a critical public health problem as the main contributor to significant long-term impairment.[1,2] Movement disorders after stroke not only reduce the activities of daily living (ADL) and affect the health of patients but also place a heavy strain on the society as a whole.[2,3] Hypofunction of the upper limbs is one of the most common types of dyskinesia following stroke, which seriously affects the patient's ADL and hampers rehabilitation progression.[4,5] In stroke rehabilitation, improving the upper extremity functional capacity remains a challenging issue.

The effectiveness of repetitive transcranial magnetic stimulation (rTMS) as stroke rehabilitation therapy has been thoroughly studied.[5,6] This technique promotes brain plasticity by providing continuous electrical pulses to the brain to alter the cortical excitability.[7] After stroke, neuroplasticity-induced cortical reconfiguration is necessary for the restoration of motor performance.[8] According to widespread consensus, low-frequency (≤1 Hz) rTMS can reduce neuronal activity and cortical excitability, whereas high-frequency (≥5 Hz) rTMS has the opposite effect.[9] The interhemispheric inhibition (IHI) model is the foundation of the current rTMS intervention method to support the improvement of upper extremity performance in stroke patients.[10] It indicates that decreased excitability of the lesioned primary motor cortex (M1) region after stroke results from "double obstacles," including the ipsilateral areas and excessive inhibition in the contralateral areas.[11] Therefore, lowering interhemispheric inhibition by minimizing the excitation of the contralesional side will improve recovery.[12] The IHI model currently serves as the foundation for recommendations regarding the application of rTMS in stroke recovery, with low-frequency rTMS to the contralesional M1 (cM1) ranked as an A-level recommendation for improving motor function.[9] Because the heterogeneity of patients is not taken into account, the clinical efficacy of this rTMS intervention strategy for poststroke motor dysfunction is limited.[9] However, for patients with severe stroke with low structural retention, using the vicariation model may be preferred, which posits that activity in the remaining network locations compensates for the loss of certain capabilities in damaged areas.[13]

In patients with severe stroke, the dorsal premotor cortex (PMd) of the contralesional hemisphere plays an impressive compensatory role in motor function recovery.[14,15] PMd has extensive connections to M1 as well as specific motor regions in the cerebral motor connections of the parietal and frontal lobes[16]. In a study on interhemispheric cPMd-iM1 interactions, Bestmann et al. used paired-coil TMS through concurrent TMS-fMRI and confirmed that conditioning cPMd pulses had a facilitative effect on ipsilesional M1.[17] This depends on the rising excitability in ipsilesional sensorimotor areas (BA4p), which maintains direct projections to spinal motoneurons and then

For peer review only

increases the motor outputs of the paralyzed upper limb.[17] Therefore, we expect that cPMd, as a potential therapeutic target, can facilitate the rehabilitation of upper extremity movements following a stroke.

To this end, this randomized controlled clinical trial aims to investigate whether the excitatory rTMS protocol over cPMd has a better effect than the inhibitory rTMS protocol over cM1. In addition to common clinical scales for evaluating upper limb motor function, diffusion tensor imaging (DTI) and functional magnetic resonance imaging (fMRI) will be used to explore changes in structural and functional connectivity, analyze the correlation between clinical function assessments and neuroimaging data, and further elucidate the neurological mechanisms underlying poststroke upper limb rehabilitation.[18] Several previous investigations have shown that cM1 inhibition does not significantly increase the rehabilitation of upper extremity movements in severe stroke patients.[15,19] As a result, we hypothesize that the excitatory rTMS protocol over cPMd might be an effective therapy for the recovery of upper extremity motor performance following severe stroke. This research may offer significant novel clues into the clinical effects of rTMS in the treatment of upper extremity impairment in stroke patients and the underlying neuroplasticity mechanisms of TMS intervention.

2 Materials and Analysis

This study is a single-blind, controlled, randomized clinical trial with two groups (A and B). Study will be carried out in the Rehabilitation Medicine Center, The First Affiliated Hospital of Nanjing Medical University, Nanjing, China. Before the trial, detailed information about the research project was provided to potential participants. After the patients voluntarily sign the informed consent form, they will be invited to participate in the trial. The improvement of adherence lies in the need for adequate communication before enrollment, so that the participants completely understand the significance and responsibilities of this study and volunteer to participate.

All eligible participants will be randomly assigned (1:1) to either group A (n=22) or group B (n=22). **Figure 1** depicts the experiment flowchart. The study protocol has been approved by the Ethics Committee of the First Affiliated Hospital of Nanjing Medical University (2020-SR-266) and registered at www.chictr.org.cn (ChiCTR2000038049).

2.1 Participants

2.1.1 Inclusion criteria

The following are the inclusion criteria:(1) ischemic stroke confirmed by computed tomography (CT) and/or MRI meets the diagnostic criteria for cerebral infarction in the Cerebrovascular Disease; (2) primary or unilateral onset or previous onset without residual neurological dysfunction; (3) stable vital signs and clear consciousness; (4) the age ranges from 40 to 70 years; (5) the course of disease ranges from 1 week to 3 months; (6) paralysis of the upper limbs; (7) FMA-UE score ≤ 22; and (8) the selected candidate or his relatives sign the informed consent form.

2.1.2 Exclusion criteria

The following are the exclusion criteria: (1) epilepsy in the previous, a family history of epilepsy, and taking medications that cause seizures; (2) the function of the urinary, cardiovascular, respiratory, and other important systems decreases or fails; (3) severe deficits in cognition and communication that impede patient participation during evaluation and therapy; (4) posterior circulation infarction; (5) TMS and fMRI-related restrictions, including a pacemaker, an artificial metal heart valve implant,

For peer review only

a drug treatment pump, an insulin pump, an aneurysm clip (other than titanium alloy, which is not paramagnetic), and a metal implant in the body; (6) severe cervical spondylosis includes severe cervical stenosis and cervical spinal instability; (7) complete occlusion of the internal carotid artery; (8) direct injuries and skull defects in the stimulation area; (9) women during pregnancy; (10) severe high fever; (11) claustrophobia; and (12) inability to cooperate with fMRI examination.

2.2 Sample size

The sample size was estimated using GPower (version 3.1.9.7). To evaluate the impact of LF-rTMS protocol over cM1 versus HF-rTMS protocol over cPMd on functional motor performance of the upper extremities evaluated utilizing FMA-UE (the primary outcome) over 2 weeks, a sample size of 42 patients (21 per group) completing the intervention will be expected to reach the statistical power of 80% at the significance level of 0.05 (two-sided test), assuming a small-to-moderate effect size of Cohen's d=0.45.[20] We will randomize 44 patients (22 in each group) to account for a 5% dropout rate.

2.3 Randomization and blinding

After the baseline assessment is completed and the inclusion/exclusion criteria are checked, the participants will be randomized into group A or B (ratio 1:1) using SPSS software to generate random sequences. This study is a single-blind study. However, the evaluators who perform the measurements will be blinded to the group to which the participants belong. Special personnel are responsible for randomization, assessment and intervention, respectively.

2.4 Intervention

All patients will receive current routine pharmacological treatments and an 80-min rehabilitation therapy session that combines physical therapy for upper limb function, neuromuscular electrical stimulation, occupational therapy, and rTMS therapy. Over the course of 2 weeks, all treatments will be administered once daily, 5 days a week (10 sessions in total). The relevant period segments of the experiment are shown in **Figure 2**.

rTMS will be applied with a CCY-I TMS stimulator (Yiruide Co., Wuhan, China) using a figure-8-shaped coil for accurate targeted treatment. The center of the coil will be tangential to the treatment plane, and the handle will be positioned at 45° from the sagittal plane. Using the surface electromyography (EMG) data that will be collected from the abductor pollicis brevis on the uninjured side, the potential difference between the highest and lowest peaks will be selected to obtain the motor-evoked potential (MEP). The minimal intensity required to generate at least 5 out of 10 MEPs in a relaxed target muscle that are >50 μV is known as the resting motor threshold (RMT).[21] The group A strategy will use rTMS (1 Hz) over cM1, 90% RMT, 120 trains of 10-s duration, 2-s inter-trial intervals, and 1200 pulses per session. The group B strategy will use rTMS (10 Hz) over cPMd, 90% RMT, 80 trains of 1.5-s duration, 10-s inter-trial intervals, and 1200 pulses per session. The coil positioning will be guided throughout the experiment using a neuronavigation system (Visor2 ANT; Germany). [22] For a period of 2 weeks, each patient will receive rTMS stimulation once each day (5 days each week).

2.5 Outcome Assessment

2.5.1 Primary Outcome

For peer review only

FMA-UE will be used to evaluate performance-based motor functions of the paralyzed upper limbs. The FMA-UE consists of 9 major items and 33 sub-items. Each item receives a rating between 0 and 3, and the total score ranges from 0 (no movement) to 66 (normal active movement). A higher score indicates lower motor impairment.[23]

2.5.2 Secondary Outcomes

2.5.2.1 Clinical and functional assessments

AMI includes shoulder abduction, elbow flexion, and pinch grip strength. The overall score of the scale is calculated by summing the scores of the three components (the highest score for each item is 33 points) and 1 point, so that a total of 100 points is reached. The score is positively correlated with the motor performance of the upper limb.[24]

FTHUE-HK includes 12 test items with 7 functional levels that evaluate upper limb function as a whole. The condition in which the patient has completed the test for this level is determined according to the requirements of each level.[25] A higher level indicates that the patients have better upper extremity motor function and a greater capacity for using the upper limbs during daily activities.

MBI[26] is a 10-item rating scale that measures the quality of general life activities. The higher the score, the more independent the patients are in their daily lives. Scores > 60 indicate a high likelihood of performing ADLs and maintaining a standard of living.

MAS is the most widely used clinical scale to assess the increase in muscle tone after central nervous system lesions, which is manifested by increased resistance of joints to passive movement. According to the MAS, possible scores range from 0, 1, 1^+, 2, 3, and 4, with 0 reflecting normal tone and 4 reflecting fixed muscle contracture.[27]

2.5.2.2 DTI and fMRI indicators

MR scanning will be performed using a 3.0T MR scanner (Discovery750W, GE Healthcare, USA) and a 24-channel head coil. In addition to T2WI scans to exclude parenchymal brain lesions and abnormalities, anatomical images will be obtained using a 3D gradient-echo pulse sequence to collect T1–weighted images in the sagittal plane. The following will be the parameters: repetition time (TR)=8.5 ms, time echo (TE)=2.52 ms, inversion time (TI)=450 ms, field of view (FOV)=256×256 mm², matrix size=256×256, flip angle=12°, image matrix=128×128, number of excitations (NEX)=1, bandwidth=31.25 kHz, 1.0-mm thick slices, and 192 slices. Data Processing Assistant Resting-State fMRI (DPARSF) software (http://rfmri.org/DPARSF) will be used to preprocess the functional imaging data. DTI determines lesion size and fractional anisotropy (FA) within the posterior limb of the internal capsule (PLIC) to assess the integrity of the corticospinal tract (CST).[28] DTI is based on an echo-planar imaging (EPI) sequence. The parameters were as follows: TR=8000 ms, TE=96 ms, image matrix=112×112, FOV=22.4×22.4 cm², b=0 and 1000 s/mm², NEX=2, slice thickness=3 mm, interslice spacing=0 mm, and acceleration factor=2. FA values and the mean FA asymmetry index (FAAI) are the leading indicators of tract integrity. FAAI=(FAunaffected−FAaffected)/(FAunaffected+FAaffected)[29]. FAAI is in the range of −1.0 to +1.0.

2.6 Statistical Analysis

2.6.1 Clinical Data Analysis

Statistical analysis will be employed using SPSS statistical software (version 22.0, Chicago, IL, United States). Patient characteristics and clinical measures will be expressed as mean and standard

For peer review only

deviation for continuous data and frequency and percentage for categorical data. We will use two-sample t-tests or Fisher's exact test to analyze the differences between the two groups for continuous and categorical data, respectively. Repeated-measures analysis of variance (ANOVA) will be used to estimate the therapeutic effect for primary (e.g., FMA-UE) and secondary outcomes (e.g., list all secondary outcomes here). Statistical significance is defined as $p<0.05$.

2.6.2 Imaging Data Analysis

DTI datasets will be processed using the PANDA toolkit (http://www.nitrc.org/projects/panda) to obtain the average FA and FAAI values of patients from the whole group.[30] The PANDA process involves three major steps: pre-processing, producing diffusion metrics, and constructing networks. The bilateral PLIC, pons, and precentral gyrus are selected as regions of interest (ROIs). Comparisons within groups will be performed using paired t-tests, and those between groups using two-sample t-tests. Structural and functional connectivity will be processed using the Infinitome software (Omniscient Neurotechnology (2020) Infinitome). The statistical significance level is set at $p<0.05$.

For the Human Connectome Project Multimodal Parcellation maps, specific topic versions will be produced using a machine-learning approach. Depending on the paired functioning connection among each participant's brain maps, it will be used to model their diagnostic classification and neuropsychological examination results. A "boosted trees" method will be applied to every case by the XGB Classifier to adapt the model. This strategy offers better predictive capabilities than using a single tree.

2.7 Safety

The intensity, frequency, and other parameter settings of rTMS will comply with the safety guidelines[31] issued by the *International Transcranial Magnetic Association* in 2009. To reduce the probability of an adverse event, the participants will be rigorously screened in accordance with the exclusion and inclusion criteria. Adverse effects and events will be closely monitored during the clinical trial. If this occurs, it will be noted in the case report form (CRF). Some incidents will be monitored, and when these incidents are inappropriate, the treatment will be discontinued, such as exacerbation of the condition, serious adverse events, poor adherence leading to a loss of follow-up, or the development of a new serious illness affecting the course of this protocol. This study is performed under the supervision of the Hospital Research Ethics Committee. Study progress and existing problems are regularly reported, and adjustments are made to overcome them in a timely manner.

3 Data Availability Statement

The data presented in this article can be obtained from the corresponding author on reasonable request.

4 Conflict of Interest

The authors declare that the research was conducted in the absence of any commercial or financial relationships that could be construed as a potential conflict of interest.

5 Author Contributions

For peer review only

TW and YS conceived and designed the study; WD, CL, XY, YZ and LF performed the study and collect materials; HD, CG and YQ analyzed the results; WD, XY and MD wrote the manuscript; YS, TW and WD helped coordinate the study and reviewed the manuscript. All authors contributed to the article and approved the submitted version.

6 Funding

This study was funded by the National Key Research &Development Program of China (2022YFC2009700) and the Nanjing Municipal Science and Technology Bureau（2019060002).

7 References

1. Allman C, Amadi U, Winkler AM, et al. Ipsilesional anodal tDCS enhances the functional benefits of rehabilitation in patients after stroke. *Sci Transl Med*. 2016;8(330):330re1. doi:10.1126/scitranslmed.aad5651

2. Hosomi K, Morris S, Sakamoto T, et al. Daily Repetitive Transcranial Magnetic Stimulation for Poststroke Upper Limb Paresis in the Subacute Period. *J Stroke Cerebrovasc Dis*. 2016;25(7):1655-1664. doi:10.1016/j.jstrokecerebrovasdis.2016.02.024

3. Turolla A, Dam M, Ventura L, et al. Virtual reality for the rehabilitation of the upper limb motor function after stroke: a prospective controlled trial. *J Neuroeng Rehabil*. 2013;10:85. doi:10.1186/1743-0003-10-85

4. Persson HC, Parziali M, Danielsson A, Sunnerhagen KS. Outcome and upper extremity function within 72 hours after first occasion of stroke in an unselected population at a stroke unit. A part of the SALGOT study. *BMC Neurol*. 2012;12:162. doi:10.1186/1471-2377-12-162

5. Fisicaro F, Lanza G, Grasso AA, et al. Repetitive transcranial magnetic stimulation in stroke rehabilitation: review of the current evidence and pitfalls. *Ther Adv Neurol Disord*. 2019;12:1756286419878317. doi:10.1177/1756286419878317

6. Murase N, Duque J, Mazzocchio R, Cohen LG. Influence of interhemispheric interactions on motor function in chronic stroke. *Ann Neurol*. 2004;55(3):400-409. doi:10.1002/ana.10848

7. Adeyemo BO, Simis M, Macea DD, Fregni F. Systematic review of parameters of stimulation, clinical trial design characteristics, and motor outcomes in non-invasive brain stimulation in stroke. *Front Psychiatry*. 2012;3:88. doi:10.3389/fpsyt.2012.00088

8. Buma F, Kwakkel G, Ramsey N. Understanding upper limb recovery after stroke. *Restor Neurol Neurosci*. 2013;31(6):707-722. doi:10.3233/RNN-130332

9. Lefaucheur JP, Aleman A, Baeken C, et al. Evidence-based guidelines on the therapeutic use of repetitive transcranial magnetic stimulation (rTMS): An update (2014-2018). *Clin Neurophysiol*. 2020;131(2):474-528. doi:10.1016/j.clinph.2019.11.002

10. Cunningham DA, Knutson JS, Sankarasubramanian V, Potter-Baker KA, Machado AG, Plow EB. Bilateral Contralaterally Controlled Functional Electrical Stimulation Reveals New Insights Into the Interhemispheric Competition Model in Chronic Stroke. *Neurorehabil Neural Repair*. 2019;33(9):707-717. doi:10.1177/1545968319863709

For peer review only

11. Yang Y, Pan H, Pan W, et al. Repetitive Transcranial Magnetic Stimulation on the Affected Hemisphere Enhances Hand Functional Recovery in Subacute Adult Stroke Patients: A Randomized Trial. *Front Aging Neurosci*. 2021;13:636184. doi:10.3389/fnagi.2021.636184

12. Dodd KC, Nair VA, Prabhakaran V. Role of the Contralesional vs. Ipsilesional Hemisphere in Stroke Recovery. *Front Hum Neurosci*. 2017;11:469. doi:10.3389/fnhum.2017.00469

13. Di Pino G, Pellegrino G, Assenza G, et al. Modulation of brain plasticity in stroke: a novel model for neurorehabilitation. *Nat Rev Neurol*. 2014;10(10):597-608. doi:10.1038/nrneurol.2014.162

14. Plow EB, Sankarasubramanian V, Cunningham DA, et al. Models to Tailor Brain Stimulation Therapies in Stroke. *Neural Plast*. 2016;2016:4071620. doi:10.1155/2016/4071620

15. Sankarasubramanian V, Machado AG, Conforto AB, et al. Inhibition versus facilitation of contralesional motor cortices in stroke: Deriving a model to tailor brain stimulation. *Clin Neurophysiol*. 2017;128(6):892-902. doi:10.1016/j.clinph.2017.03.030

16. Kantak SS, Stinear JW, Buch ER, Cohen LG. Rewiring the brain: potential role of the premotor cortex in motor control, learning, and recovery of function following brain injury. *Neurorehabil Neural Repair*. 2012;26(3):282-292. doi:10.1177/1545968311420845

17. Bestmann S, Swayne O, Blankenburg F, et al. The role of contralesional dorsal premotor cortex after stroke as studied with concurrent TMS-fMRI. *J Neurosci*. 2010;30(36):11926-11937. doi:10.1523/JNEUROSCI.5642-09.2010

18. Orihuela-Espina F, Fernández del Castillo I, Palafox L, et al. Neural reorganization accompanying upper limb motor rehabilitation from stroke with virtual reality-based gesture therapy. *Top Stroke Rehabil*. 2013;20(3):197-209. doi:10.1310/tsr2003-197

19. Seniów J, Bilik M, Leśniak M, Waldowski K, Iwański S, Członkowska A. Transcranial magnetic stimulation combined with physiotherapy in rehabilitation of poststroke hemiparesis: a randomized, double-blind, placebo-controlled study. *Neurorehabil Neural Repair*. 2012;26(9):1072-1079. doi:10.1177/1545968312445635

20. Guan YZ, Li J, Zhang XW, et al. Effectiveness of repetitive transcranial magnetic stimulation (rTMS) after acute stroke: A one-year longitudinal randomized trial. *CNS Neurosci Ther*. 2017;23(12):940-946. doi:10.1111/cns.12762

21. Rossini PM, Burke D, Chen R, et al. Non-invasive electrical and magnetic stimulation of the brain, spinal cord, roots and peripheral nerves: Basic principles and procedures for routine clinical and research application. An updated report from an I.F.C.N. Committee. *Clinical Neurophysiology*. 2015;126(6):1071-1107. doi:10.1016/j.clinph.2015.02.001

22. Niérat MC, Similowski T, Lamy JC. Does trans-spinal direct current stimulation alter phrenic motoneurons and respiratory neuromechanical outputs in humans? A double-blind, sham-controlled, randomized, crossover study. *J Neurosci*. 2014;34(43):14420-14429. doi:10.1523/JNEUROSCI.1288-14.2014

For peer review only

23. Duncan PW, Propst M, Nelson SG. Reliability of the Fugl-Meyer assessment of sensorimotor recovery following cerebrovascular accident. *Phys Ther*. 1983;63(10):1606-1610. doi:10.1093/ptj/63.10.1606

24. Lang CE, Bland MD, Bailey RR, Schaefer SY, Birkenmeier RL. Assessment of upper extremity impairment, function, and activity after stroke: foundations for clinical decision making. *J Hand Ther*. 2013;26(2):104-114;quiz 115. doi:10.1016/j.jht.2012.06.005

25. Bai Y, Liu F, Zhang H. Artificial Intelligence Limb Rehabilitation System on Account of Virtual Reality Technology on Long-Term Health Management of Stroke Patients in the Context of the Internet. *Comput Math Methods Med*. 2022;2022:2688003. doi:10.1155/2022/2688003

26. Shah S, Vanclay F, Cooper B. Improving the sensitivity of the Barthel Index for stroke rehabilitation. *J Clin Epidemiol*. 1989;42(8):703-709. doi:10.1016/0895-4356(89)90065-6

27. Meseguer-Henarejos AB, Sánchez-Meca J, López-Pina JA, Carles-Hernández R. Inter- and intra-rater reliability of the Modified Ashworth Scale: a systematic review and meta-analysis. *Eur J Phys Rehabil Med*. 2018;54(4):576-590. doi:10.23736/S1973-9087.17.04796-7

28. Bradnam LV, Stinear CM, Barber PA, Byblow WD. Contralesional hemisphere control of the proximal paretic upper limb following stroke. *Cereb Cortex*. 2012;22(11):2662-2671. doi:10.1093/cercor/bhr344

29. Wilde EA, McCauley SR, Chu Z, et al. Diffusion tensor imaging of hemispheric asymmetries in the developing brain. *J Clin Exp Neuropsychol*. 2009;31(2):205-218. doi:10.1080/13803390802098118

30. Cui Z, Zhong S, Xu P, He Y, Gong G. PANDA: a pipeline toolbox for analyzing brain diffusion images. *Front Hum Neurosci*. 2013;7:42. doi:10.3389/fnhum.2013.00042

31. Rossi S, Hallett M, Rossini PM, Pascual-Leone A, Safety of TMS Consensus Group. Safety, ethical considerations, and application guidelines for the use of transcranial magnetic stimulation in clinical practice and research. *Clin Neurophysiol*. 2009;120(12):2008-2039. doi:10.1016/j.clinph.2009.08.016

Figure 1. Experiment flowchart.

FMA-UE, Fugl-Meyer Assessment of Upper Extremity; AMI, arm sub-score of the Motricity Index; FTHUE-HK, Hong Kong edition of Functional Test for the Hemiplegic Upper Extremity; MBI, Modified Barthel Index; MAS, Modified Ashworth Scale; FA, fractional anisotropy; FAAI, FA asymmetry index; rTMS, repetitive transcranial magnetic stimulation.

Figure 2. Schedule of participant enrollment, interventions, and assessments.

LF-rTMS, low-frequency repetitive transcranial magnetic stimulation; HF-rTMS, high-frequency repetitive transcranial magnetic stimulation; FMA-UE, Fugl-Meyer Assessment of Upper Extremity; AMI, arm sub-score of the Motricity Index; FTHUE-HK, Hong Kong edition of Functional Test for the Hemiplegic Upper Extremity; MBI, Modified Barthel Index; MAS, Modified Ashworth Scale; fMRI, functional magnetic resonance imaging.

For peer review only

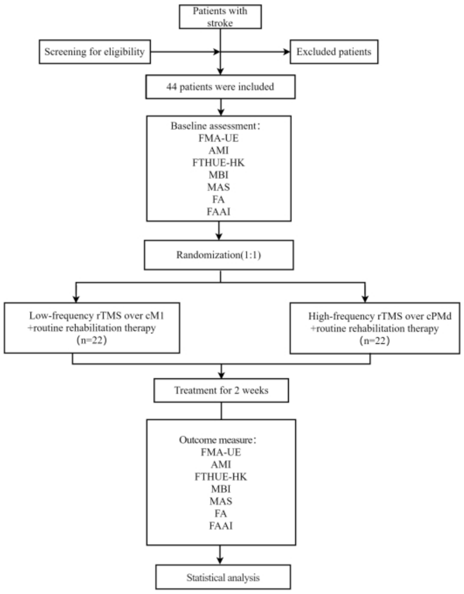

Figure1.Experiment flowchart.

297x209mm (300 x 300 DPI)

	Study Period			
	Enrollment	Allocation	Post-allocation	
**TIME POINT**	W-1	0	W1	W2
**ENROLLMENT:**				
Eligibility screen	×			
Informed consent	×			
Randomization	×			
Allocation		×		
**INTERVENTIONS:**				
LF-rTMS protocol over cM1			←	→
HF-rTMS protocol over cPMd			←	→
**ASSESSMENTS:**				
FMA-UE	×			×
AMI	×			×
FTHUE-HK	×			×
MBI	×			×
MAS	×			×
fMRI	×			×

Figure2.Schedule of participant enrollment, interventions, and assessments

337x259mm (144 x 144 DPI)

STANDARD PROTOCOL ITEMS: RECOMMENDATIONS FOR INTERVENTIONAL TRIALS

SPIRIT 2013 Checklist: Recommended items to address in a clinical trial protocol and related documents*

Section/item	Item No	Description
**Administrative information**		
Title	1	Descriptive title identifying the study design, population, interventions, and, if applicable, trial acronym ☑
Trial registration	2a	Trial identifier and registry name. If not yet registered, name of intended registry ☑
	2b	All items from the World Health Organization Trial Registration Data Set ☑
Protocol version	3	Date and version identifier ☑
Funding	4	Sources and types of financial, material, and other support ☑
Roles and responsibilities	5a	Names, affiliations, and roles of protocol contributors ☑
	5b	Name and contact information for the trial sponsor ☑
	5c	Role of study sponsor and funders, if any, in study design; collection, management, analysis, and interpretation of data; writing of the report; and the decision to submit the report for publication, including whether they will have ultimate authority over any of these activities ☑
	5d	Composition, roles, and responsibilities of the coordinating centre, steering committee, endpoint adjudication committee, data management team, and other individuals or groups overseeing the trial, if applicable (see Item 21a for data monitoring committee) ☑
**Introduction**		
Background and rationale	6a	Description of research question and justification for undertaking the trial, including summary of relevant studies (published and unpublished) examining benefits and harms for each intervention ☑
	6b	Explanation for choice of comparators ☑
Objectives	7	Specific objectives or hypotheses ☑
Trial design	8	Description of trial design including type of trial (eg, parallel group, crossover, factorial, single group), allocation ratio, and framework (eg, superiority, equivalence, noninferiority, exploratory) ☑

For peer review only

Methods: Participants, interventions, and outcomes

Study setting	9	Description of study settings (eg, community clinic, academic hospital) and list of countries where data will be collected. Reference to where list of study sites can be obtained	☑
Eligibility criteria	10	Inclusion and exclusion criteria for participants. If applicable, eligibility criteria for study centres and individuals who will perform the interventions (eg, surgeons, psychotherapists)	☑
Interventions	11a	Interventions for each group with sufficient detail to allow replication, including how and when they will be administered	☑
	11b	Criteria for discontinuing or modifying allocated interventions for a given trial participant (eg, drug dose change in response to harms, participant request, or improving/worsening disease)	☑
	11c	Strategies to improve adherence to intervention protocols, and any procedures for monitoring adherence (eg, drug tablet return, laboratory tests)	☑
	11d	Relevant concomitant care and interventions that are permitted or prohibited during the trial	☑
Outcomes	12	Primary, secondary, and other outcomes, including the specific measurement variable (eg, systolic blood pressure), analysis metric (eg, change from baseline, final value, time to event), method of aggregation (eg, median, proportion), and time point for each outcome. Explanation of the clinical relevance of chosen efficacy and harm outcomes is strongly recommended	☑
Participant timeline	13	Time schedule of enrolment, interventions (including any run-ins and washouts), assessments, and visits for participants. A schematic diagram is highly recommended (see Figure)	☑
Sample size	14	Estimated number of participants needed to achieve study objectives and how it was determined, including clinical and statistical assumptions supporting any sample size calculations	☑
Recruitment	15	Strategies for achieving adequate participant enrolment to reach target sample size	☑

Methods: Assignment of interventions (for controlled trials)

Allocation:

Sequence generation	16a	Method of generating the allocation sequence (eg, computer-generated random numbers), and list of any factors for stratification. To reduce predictability of a random sequence, details of any planned restriction (eg, blocking) should be provided in a separate document that is unavailable to those who enrol participants or assign interventions	☑

Allocation concealment mechanism	16b	Mechanism of implementing the allocation sequence (eg, central telephone; sequentially numbered, opaque, sealed envelopes), describing any steps to conceal the sequence until interventions are assigned ☑
Implementation	16c	Who will generate the allocation sequence, who will enrol participants, and who will assign participants to interventions ☑
Blinding (masking)	17a	Who will be blinded after assignment to interventions (eg, trial participants, care providers, outcome assessors, data analysts), and how ☑
	17b	If blinded, circumstances under which unblinding is permissible, and procedure for revealing a participant's allocated intervention during the trial ☑

Methods: Data collection, management, and analysis

Data collection methods	18a	Plans for assessment and collection of outcome, baseline, and other trial data, including any related processes to promote data quality (eg, duplicate measurements, training of assessors) and a description of study instruments (eg, questionnaires, laboratory tests) along with their reliability and validity, if known. Reference to where data collection forms can be found, if not in the protocol ☑
	18b	Plans to promote participant retention and complete follow-up, including list of any outcome data to be collected for participants who discontinue or deviate from intervention protocols ☑
Data management	19	Plans for data entry, coding, security, and storage, including any related processes to promote data quality (eg, double data entry; range checks for data values). Reference to where details of data management procedures can be found, if not in the protocol ☑
Statistical methods	20a	Statistical methods for analysing primary and secondary outcomes. Reference to where other details of the statistical analysis plan can be found, if not in the protocol ☑
	20b	Methods for any additional analyses (eg, subgroup and adjusted analyses) ☒ Methods for any additional analyses were not employed in this study.
	20c	Definition of analysis population relating to protocol non-adherence (eg, as randomised analysis), and any statistical methods to handle missing data (eg, multiple imputation) ☑

Methods: Monitoring

For peer review only - http://bmjopen.bmj.com/site/about/guidelines.xhtml

Data monitoring	21a	Composition of data monitoring committee (DMC); summary of its role and reporting structure; statement of whether it is independent from the sponsor and competing interests; and reference to where further details about its charter can be found, if not in the protocol. Alternatively, an explanation of why a DMC is not needed ☑
	21b	Description of any interim analyses and stopping guidelines, including who will have access to these interim results and make the final decision to terminate the trial ☑
Harms	22	Plans for collecting, assessing, reporting, and managing solicited and spontaneously reported adverse events and other unintended effects of trial interventions or trial conduct ☑
Auditing	23	Frequency and procedures for auditing trial conduct, if any, and whether the process will be independent from investigators and the sponsor ☑
**Ethics and dissemination**		
Research ethics approval	24	Plans for seeking research ethics committee/institutional review board (REC/IRB) approval ☑
Protocol amendments	25	Plans for communicating important protocol modifications (eg, changes to eligibility criteria, outcomes, analyses) to relevant parties (eg, investigators, REC/IRBs, trial participants, trial registries, journals, regulators) ☑
Consent or assent	26a	Who will obtain informed consent or assent from potential trial participants or authorised surrogates, and how (see Item 32) ☑
	26b	Additional consent provisions for collection and use of participant data and biological specimens in ancillary studies, if applicable ☒This content wasn't involved in the study.
Confidentiality	27	How personal information about potential and enrolled participants will be collected, shared, and maintained in order to protect confidentiality before, during, and after the trial ☑
Declaration of interests	28	Financial and other competing interests for principal investigators for the overall trial and each study site ☑
Access to data	29	Statement of who will have access to the final trial dataset, and disclosure of contractual agreements that limit such access for investigators ☑
Ancillary and post-trial care	30	Provisions, if any, for ancillary and post-trial care, and for compensation to those who suffer harm from trial participation ☑

For peer review only - http://bmjopen.bmj.com/site/about/guidelines.xhtml

Dissemination policy	31a	Plans for investigators and sponsor to communicate trial results to participants, healthcare professionals, the public, and other relevant groups (eg, via publication, reporting in results databases, or other data sharing arrangements), including any publication restrictions ☑
	31b	Authorship eligibility guidelines and any intended use of professional writers ☑
	31c	Plans, if any, for granting public access to the full protocol, participant-level dataset, and statistical code ☑
**Appendices**		
Informed consent materials	32	Model consent form and other related documentation given to participants and authorised surrogates ☑
Biological specimens	33	Plans for collection, laboratory evaluation, and storage of biological specimens for genetic or molecular analysis in the current trial and for future use in ancillary studies, if applicable ☒ There weren't plans for collection, laboratory evaluation, or storage of biological specimens in this study.

*It is strongly recommended that this checklist be read in conjunction with the SPIRIT 2013 Explanation & Elaboration for important clarification on the items. Amendments to the protocol should be tracked and dated. The SPIRIT checklist is copyrighted by the SPIRIT Group under the Creative Commons "Attribution-NonCommercial-NoDerivs 3.0 Unported" license.

For peer review only

For peer review only - http://bmjopen.bmj.com/site/about/guidelines.xhtml

BMJ Open

Effects of repetitive transcranial magnetic stimulation over the contralesional dorsal premotor cortex on upper limb function in severe ischemic stroke: study protocol for a randomized controlled trial

Journal:	*BMJ Open*
Manuscript ID	bmjopen-2023-074037.R1
Article Type:	Protocol
Date Submitted by the Author:	10-Jul-2023
Complete List of Authors:	Dai, Wenjun; Jiangsu Province Hospital and Nanjing Medical University First Affiliated Hospital, Rehabilitation Medicine Center<br>Liu, Canhuan; Nanjing Medical University, School of Rehabilitation Medicine<br>Yang, Xi; Nanjing Medical University, School of Rehabilitation Medicine<br>Ding, Hongyuan; Jiangsu Province Hospital and Nanjing Medical University First Affiliated Hospital, Department of Radiology<br>Guo, Chuan; Jiangsu Province Hospital and Nanjing Medical University First Affiliated Hospital, Rehabilitation Medicine Center<br>Zhu, Yi; Jiangsu Province Hospital and Nanjing Medical University First Affiliated Hospital, Rehabilitation Medicine Center<br>Dong, Manyu; Nanjing Medical University, School of Rehabilitation Medicine<br>Qian, Yilun; Nanjing Medical University, School of Rehabilitation Medicine<br>Fang, Lu; Jiangsu Province Hospital and Nanjing Medical University First Affiliated Hospital, Rehabilitation Medicine Center<br>Wang, Tong; Jiangsu Province Hospital and Nanjing Medical University First Affiliated Hospital, Rehabilitation Medicine Center<br>Shen, Ying; Jiangsu Province Hospital and Nanjing Medical University First Affiliated Hospital, Rehabilitation Medicine Center
**Primary Subject Heading**:	Rehabilitation medicine
Secondary Subject Heading:	Neurology
Keywords:	Stroke < NEUROLOGY, REHABILITATION MEDICINE, Clinical trials < THERAPEUTICS

SCHOLARONE™
Manuscripts

Effects of repetitive transcranial magnetic stimulation over the contralesional dorsal premotor cortex on upper limb function in severe ischemic stroke: study protocol for a randomized controlled trial

Wenjun Dai[1#], Canhuan Liu[2#], Xi Yang[2#], Hongyuan Ding[3#], Chuan Guo[1], Yi Zhu[1], Manyu Dong[2], Yilun Qian[2], Lu Fang[1], Tong Wang[1*], Ying Shen[1*]

[1] Rehabilitation Medicine Center, The First Affiliated Hospital of Nanjing Medical University, Nanjing, China

[2] School of Rehabilitation Medicine, Nanjing Medical University, Nanjing, China

[3] Department of Radiology, The First Affiliated Hospital of Nanjing Medical University, Nanjing, China

[#]These authors have contributed equally to this work and share first authorship

*** Correspondence:**
Ying Shen
shenying@njmu.edu.cn
Tong Wang
wangtong60621@163.com

For peer review only

Abstract

Introduction: Repetitive transcranial magnetic stimulation (rTMS) is an evidence-based treatment widely recommended to promote hand motor recovery after ischemic stroke. However, the therapeutic efficacy of rTMS over the motor cortex in stroke patients is currently restricted and heterogeneous. This study aimed to determine whether excitatory rTMS over the contralesional dorsal premotor cortex (cPMd) facilitates the functional recovery of the upper limbs during the post-acute stage of severe ischemic stroke.

Methods and Analysis: This study will be conducted as a single-blind, controlled, randomized study, in which 44 patients with poststroke hemiplegia with a course of disease ranging from 1 week to 3 months and Fugl-Meyer upper limb score ≤22 will be enrolled. The study participants will be randomly assigned to groups A (n=22) and B (n=22). The two groups are based on routine rehabilitation training and drug treatment; group A will be treated with low-frequency (1 Hz) rTMS over the contralesional primary motor cortex (cM1), and group B will be treated with high-frequency rTMS (10 Hz) over cPMd. For 2 weeks, rTMS will be administered once a day, 5 days a week. The primary outcome is the Fugl–Meyer assessment of the upper limb. The secondary outcomes include the arm sub-score of the motricity index (AMI), Hong Kong edition of Functional Test for the Hemiplegic Upper Extremity (FTHUE-HK), Modified Barthel Index (MBI), and Modified Ashworth Scale (MAS) score of the paralyzed pectoralis major (PM) and biceps brachii (BI). Furthermore, data of diffusion tensor imaging (DTI) and functional magnetic resonance imaging (fMRI) will be collected. These outcomes will be assessed before and after the completion of the intervention.

Clinical Trial registration number Chinese Clinical Trial Registry ChiCTR2000038049

Strengths and limitations of this study

1. This will be a single-blinded, controlled, randomized clinical study to compare high-frequency rTMS over cPMd and low-frequency rTMS over cM1 in severe ischemic stroke.
2. We will use neuronavigation to position the site of cPMd and cM1 stimulation.
3. Additionally, various outcome measures, including clinical function and functional magnetic resonance imaging, will be included to investigate the therapeutic effects of rTMS and its underlying neuroplasticity mechanisms.
4. Because of low-frequency rTMS to the cM1 ranked as an A-level recommendation for improving motor function after stroke, no blank control group undergoing sham TMS will be set up.
5. Given the nature of TMS interventions, blinding operators is not feasible.

1 Introduction

Ischemic stroke has emerged as a critical public health problem as the main contributor to significant long-term impairment.[1,2] Movement disorders after ischemic stroke not only reduce the activities of daily living (ADL) and affect the health of patients but also place a heavy strain on the society as a whole.[2,3] Hypofunction of the upper limbs is one of the most common types of dyskinesia following ischemic stroke, which seriously affects the patient's ADL and hampers rehabilitation progression.[4,5] In ischemic stroke rehabilitation, improving the upper extremity functional capacity remains a challenging issue.

The effectiveness of repetitive transcranial magnetic stimulation (rTMS) as ischemic stroke rehabilitation therapy has been thoroughly studied.[5,6] This technique promotes brain plasticity by providing continuous magnetic pulses to the brain .[7] After stroke, neuroplasticity-induced cortical reconfiguration is necessary for the restoration of motor performance.[8] According to widespread consensus, low-frequency (≤1 Hz) rTMS can reduce neuronal activity and cortical excitability, whereas high-frequency (≥5 Hz) rTMS has the opposite effect.[9] The interhemispheric inhibition (IHI) model is the foundation of the current rTMS intervention method to support the improvement of upper extremity performance in stroke patients.[10] It indicates that decreased excitability of the lesioned primary motor cortex (M1) region after stroke results from “double obstacles,” including the ipsilateral areas and excessive inhibition in the contralateral areas.[11] Therefore, lowering interhemispheric inhibition by minimizing the excitation of the contralesional side will improve recovery.[12] The IHI model currently serves as the foundation for recommendations regarding the application of rTMS in stroke recovery, with low-frequency rTMS to the contralesional M1 (cM1) ranked as an A-level recommendation for improving motor function.[9] Because the heterogeneity of patients is not taken into account, the clinical efficacy of this rTMS intervention strategy for poststroke motor dysfunction is limited.[9] However, for patients with severe stroke with low structural retention, using the vicariation model may be preferred, which posits that activity in the remaining network locations compensates for the loss of certain capabilities in damaged areas.[13]

In patients with severe ischemic stroke, the dorsal premotor cortex (PMd) of the contralesional hemisphere plays an impressive compensatory role in motor function recovery.[14,15] PMd has extensive connections to M1 as well as specific motor regions in the cerebral motor connections of the parietal and frontal lobes.[16] Vishwanath et al. found that the novel approach of cPMd facilitation (5Hz rTMS) resulted in more improvement than cM1 inhibition (1Hz rTMS) in severely impaired stroke patients by alleviating interhemispheric competition inflicted on weak iM1.[15] cPMd reduces inhibition of weak iM1 and provides ipsilateral access (uncrossed corticospinal and brainstem-mediated reticulospinal) to the paralyzed limb to aid recovery.[14,17] Lotze proposed an inverse correlation between dorsal premotor cortex activation and ipsilateral corticospinal tract integrity, verifying the potential importance of contralateral dorsal premotor cortex for good motor

performance.[18] In a study on interhemispheric cPMd-iM1 interactions, Bestmann et al. used paired-coil TMS through concurrent TMS-fMRI and confirmed that conditioning cPMd pulses had a facilitative effect on ipsilesional M1.[19] This depends on the rising excitability in ipsilesional sensorimotor areas (BA4p), which maintains direct projections to spinal motoneurons and then increases the motor outputs of the paralyzed upper limb.[19] Therefore, we expect that cPMd, as a potential therapeutic target, can facilitate the rehabilitation of upper extremity movements following a ischemic stroke.

To this end, this randomized controlled clinical trial aims to investigate whether the excitatory rTMS protocol over cPMd has a better effect than the inhibitory rTMS protocol over cM1 to compensate for the limitations of its inconsistent therapeutic effects. In addition to common clinical scales for evaluating upper limb motor function, diffusion tensor imaging (DTI) and functional magnetic resonance imaging (fMRI) will be used to explore changes in structural and functional connectivity, analyze the correlation between clinical function assessments and neuroimaging data, and further elucidate the neurological mechanisms underlying poststroke upper limb rehabilitation.[20] Several previous investigations have shown that cM1 inhibition does not significantly increase the rehabilitation of upper extremity movements in severe stroke patients.[15,21] As a result, we hypothesize that the excitatory rTMS protocol over cPMd might be an effective therapy for the recovery of upper extremity motor performance following severe ischemic stroke. This research may offer significant novel clues into the clinical effects of rTMS in the treatment of upper extremity impairment in ischemic stroke patients and the underlying neuroplasticity mechanisms of TMS intervention.

2 Materials and Analysis

This study is a single-blind, controlled, randomized clinical trial with two groups (A and B). Study will be carried out in the Rehabilitation Medicine Center, The First Affiliated Hospital of Nanjing Medical University, Nanjing, China. Before the trial, detailed information about the research project was provided to potential participants. After the patients voluntarily sign the informed consent form, they will be invited to participate in the trial. The improvement of adherence lies in the need for adequate communication before enrollment, so that the participants completely understand the significance and responsibilities of this study and volunteer to participate.

All eligible participants will be randomly assigned (1:1) to either group A (n=22) or group B (n=22). In consideration of research purposes, ethical perspectives, feasibility, and practicality, etc., this study was not designed with a placebo control group. **Figure 1** depicts the experiment flowchart. The study protocol has been approved by the Ethics Committee of the First Affiliated Hospital of Nanjing Medical University (2020-SR-266) and registered on Chinese Clinical Trial Registry (ChiCTR2000038049, website: http:// www.chictr.org.cn).

2.1 Participants

2.1.1 Inclusion criteria

The following are the inclusion criteria:(1) ischemic stroke confirmed by computed tomography (CT) and/or MRI meets the diagnostic criteria for cerebral infarction in the Cerebrovascular Disease; (2) primary or unilateral onset or previous onset without residual neurological dysfunction; (3) stable vital signs and clear consciousness; (4) the age ranges from 40 to 70 years; (5) the course of disease ranges from 1 week to 3 months; (6) paralysis of the upper limbs; (7) FMA-UE score $\leq$ 22; and (8) the selected candidate or his relatives sign the informed consent form.

For peer review only

For peer review only - http://bmjopen.bmj.com/site/about/guidelines.xhtml

2.1.2 Exclusion criteria

The following are the exclusion criteria: (1) epilepsy in the previous, a family history of epilepsy, and taking medications that cause seizures; (2) the function of the urinary, cardiovascular, respiratory, and other important systems decreases or fails; (3) severe deficits in cognition and communication that impede patient participation during evaluation and therapy; (4) posterior circulation infarction; (5) TMS and fMRI-related restrictions, including a pacemaker, an artificial metal heart valve implant, a drug treatment pump, an insulin pump, an aneurysm clip (other than titanium alloy, which is not paramagnetic), and a metal implant in the body; (6) severe cervical spondylosis includes severe cervical stenosis and cervical spinal instability; (7) complete occlusion of the internal carotid artery; (8) direct injuries and skull defects in the stimulation area; (9) women during pregnancy; (10) severe high fever; (11) claustrophobia; and (12) inability to cooperate with fMRI examination.

2.2 Sample size

The sample size was estimated using GPower (version 3.1.9.7). To evaluate the impact of LF-rTMS protocol over cM1 versus HF-rTMS protocol over cPMd on functional motor performance of the upper extremities evaluated utilizing FMA-UE (the primary outcome) over 2 weeks, a sample size of 42 patients (21 per group) completing the intervention will be expected to reach the statistical power of 80% at the significance level of 0.05 (two-sided test), assuming a small-to-moderate effect size of Cohen's d=0.45.[22] We will randomize 44 patients (22 in each group) to account for a 5% dropout rate.

2.3 Randomization and blinding

After the baseline assessment is completed and the inclusion/exclusion criteria are checked, the participants will be randomized into group A or B (ratio 1:1) using SPSS software to generate random sequences. The stratification criterion is the course duration of the disease, with two strata: 1 month and 2-3 months. This study is a single-blind study. However, the evaluators who perform the measurements will be blinded to the group to which the participants belong. Special personnel are responsible for randomization, assessment and intervention, respectively.

2.4 Intervention

All patients will receive current routine pharmacological treatments and an 80-min rehabilitation therapy session that combines physical therapy for upper limb function, neuromuscular electrical stimulation, occupational therapy, and rTMS therapy. Over the course of 2 weeks, all treatments will be administered once daily, 5 days a week (10 sessions in total). The relevant period segments of the experiment are shown in **Figure 2**.

rTMS will be applied with a CCY-I TMS stimulator (Yiruide Co., Wuhan, China) using a figure-8-shaped coil for accurate targeted treatment. The center of the coil will be tangential to the treatment plane, and the handle will be positioned at 45° from the sagittal plane. Using the surface electromyography (EMG) data that will be collected from the abductor pollicis brevis on the uninjured side, the potential difference between the highest and lowest peaks will be selected to obtain the motor-evoked potential (MEP). The minimal intensity required to generate at least 5 out of 10 MEPs in a relaxed target muscle that are >50 μV is known as the resting motor threshold (RMT).[23] The group A strategy will use rTMS (1 Hz) over cM1, 90% RMT, 120 trains of 10-s duration, 2-s inter-trial intervals, 1200 pulses per session, and a 23-min, 58-s total duration. The group B strategy will use rTMS (10 Hz) over cPMd, 90% RMT, 80 trains of 1.5-s duration, 10-s inter-trial intervals, 1200 pulses per session, and a 15-min, 10-s total duration. The coil positioning

For peer review only

will be guided throughout the experiment using a neuronavigation system (Visor2 ANT; Germany).[24] For a period of 2 weeks, each patient will receive rTMS stimulation once each day (5 days each week).

2.5 Outcome Assessment

2.5.1 Primary Outcome

FMA-UE will be used to evaluate performance-based motor functions of the paralyzed upper limbs. The FMA-UE consists of 9 major items and 33 sub-items. Each item receives a rating between 0 and 3, and the total score ranges from 0 (no movement) to 66 (normal active movement). A higher score indicates lower motor impairment.[25]

2.5.2 Secondary Outcomes

2.5.2.1 Clinical and functional assessments

AMI includes shoulder abduction, elbow flexion, and pinch grip strength. The overall score of the scale is calculated by summing the scores of the three components (the highest score for each item is 33 points) and 1 point, so that a total of 100 points is reached. The score is positively correlated with the motor performance of the upper limb.[26]

FTHUE-HK includes 12 test items with 7 functional levels that evaluate upper limb function as a whole. The condition in which the patient has completed the test for this level is determined according to the requirements of each level.[27] A higher level indicates that the patients have better upper extremity motor function and a greater capacity for using the upper limbs during daily activities.

MBI[28] is a 10-item rating scale that measures the quality of general life activities. The higher the score, the more independent the patients are in their daily lives. Scores > 60 indicate a high likelihood of performing ADLs and maintaining a standard of living.

MAS is the most widely used clinical scale to assess the increase in muscle tone after central nervous system lesions, which is manifested by increased resistance of joints to passive movement. According to the MAS, possible scores range from 0, 1, 1^+, 2, 3, and 4, with 0 reflecting normal tone and 4 reflecting fixed muscle contracture.[29]

2.5.2.2 DTI and fMRI indicators

MR scanning will be performed using a 3.0T MR scanner (Discovery750W, GE Healthcare, USA) and a 24-channel head coil. In addition to T2WI scans to exclude parenchymal brain lesions and abnormalities, anatomical images will be obtained using a 3D gradient-echo pulse sequence to collect T1–weighted images in the sagittal plane. The following will be the parameters: repetition time (TR)=8.5 ms, time echo (TE)=2.52 ms, inversion time (TI)=450 ms, field of view (FOV)=256×256 mm^2, matrix size=256×256, flip angle=12°, image matrix=128×128, number of excitations (NEX)=1, bandwidth=31.25 kHz, 1.0-mm thick slices, and 192 slices. Data Processing Assistant Resting-State fMRI (DPARSF) software (http://rfmri.org/DPARSF) will be used to preprocess the functional imaging data. DTI determines lesion size and fractional anisotropy (FA) within the posterior limb of the internal capsule (PLIC) to assess the integrity of the corticospinal tract (CST).[30] DTI is based on an echo-planar imaging (EPI) sequence. The parameters were as follows: TR=8000 ms, TE=96 ms, image matrix=112×112, FOV=22.4×22.4 cm^2, b=0 and 1000 s/mm^2, NEX=2, slice thickness=3 mm, interslice spacing=0 mm, and acceleration factor=2. FA values and the mean FA asymmetry index (FAAI) are the leading indicators of tract integrity.

For peer review only - http://bmjopen.bmj.com/site/about/guidelines.xhtml

FAAI=(FAunaffected−FAaffected)/(FAunaffected+FAaffected)[31]. FAAI is in the range of −1.0 to +1.0.

2.6 Statistical Analysis

2.6.1 Clinical Data Analysis

Statistical analysis will be employed using SPSS statistical software (version 22.0, Chicago, IL, United States). Prior to entering data for descriptive statistics, the Shapiro-Wilk test will be used to verify normal probability. Data from the normal distribution will be given as mean and standard deviation, but data from the non-normal distribution will be expressed as medians with interquartile ranges. Categorical variables will be described as a function of frequency as a percentage. Demographic characteristics and baseline variables will be assessed for between-group differences using statistical tests appropriate for the data type. Specifically, independent sample t-tests or nonparametric Mann-Whitney tests will be used for continuous variables, while chi-square or Fisher's exact tests will be employed for categorical variables. Repeated-measures analysis of variance (ANOVA) will be used to estimate the therapeutic effect for primary (e.g., FMA-UE) and secondary outcomes (e.g., list all secondary outcomes here). Statistical significance is defined as $p<0.05$. The primary analysis will employ an intention-to-treat method to address non-adherence. The absence of primary outcome data will be addressed by utilizing baseline data as a reference point and conducting additional sensitivity analyses. The utilization of a randomized design precludes the use of multivariable models for confounding adjustment. Consequently, the absence of covariates will have no impact on the primary analysis.

2.6.2 Imaging Data Analysis

DTI datasets will be processed using the PANDA toolkit (http://www.nitrc.org/projects/panda) to obtain the average FA and FAAI values of patients from the whole group.[32] The PANDA process involves three major steps: pre-processing, producing diffusion metrics, and constructing networks. The bilateral PLIC, pons, and precentral gyrus are selected as regions of interest (ROIs). Comparisons within groups will be performed using paired t-tests, and those between groups using two-sample t-tests. Structural and functional connectivity will be processed using the Infinitome software (Omniscient Neurotechnology (2020) Infinitome). The statistical significance level is set at $p<0.05$.

For the Human Connectome Project Multimodal Parcellation maps, specific topic versions will be produced using a machine-learning approach. Depending on the paired functioning connection among each participant's brain maps, it will be used to model their diagnostic classification and neuropsychological examination results. A “boosted trees” method will be applied to every case by the XGB Classifier to adapt the model. This strategy offers better predictive capabilities than using a single tree.

2.7 Data collection, management and monitoring

Data collection will be conducted by highly skilled attending physicians, therapists, and radiologists who are independent of the grouping and treatment procedures. They will promptly input the gathered data into the designated case report form (CRF). Subsequently, a designated individual will

For peer review only

digitally capture all the data while ensuring the use of unique identifiers to safeguard patient privacy and data security.

The data monitoring committee (DMC) will verify whether the conduct, generation, recording, and reporting of the clinical trial comply with the regulatory requirements of the protocol, based on the monitoring plan and written standard operating procedures. By regularly reviewing the collected data, including checking the data entry and storage against predefined standards and guidelines, they examine the consistency, logic, and validity of the data. They compare the entered data with the source data to confirm its accuracy and consistency. If any anomalies or potential issues are identified, the DMC will promptly investigate and correct them. They may communicate with data collectors, researchers, or other relevant parties to clarify and resolve issues, ensuring the validity and reliability of the data. The goal of data monitoring is to minimize data errors and biases, and ensure data quality to support accurate data analysis and reliable research conclusions.

2.8 Harms

The intensity, frequency, and other parameter settings of rTMS will comply with the safety guidelines[33] issued by the *International Transcranial Magnetic Association* in 2009. To reduce the probability of an adverse event, the participants will be rigorously screened in accordance with the exclusion and inclusion criteria. Adverse effects and events will be closely monitored during the clinical trial. If this occurs, it will be noted in the CRF. Some incidents will be monitored, and when these incidents are inappropriate, the treatment will be discontinued, such as exacerbation of the condition, serious adverse events, poor adherence leading to a loss of follow-up, or the development of a new serious illness affecting the course of this protocol. This study is performed under the supervision of the Hospital Research Ethics Committee. Study progress and existing problems are semi-annually reported, and adjustments are made to overcome them in a timely manner.

3 Ethics and dissemination

3.1 Research ethics approval

This study has been approved by the Ethics Committee of First Affiliated Hospital of Nanjing Medical University(2020-SR-266) .

3.2 Protocol amendments

The modifications to the protocol will be determined through in-depth discussions and careful deliberations by the Hospital Research Ethics Committee. Once the amendments receive approval from the Chinese Clinical Trial Registry, the modified protocol will be implemented for the study. The current version of the protocol is V5.0 (date: June 28, 2021).

3.3 Consent or assent

The researchers diligently provide comprehensive information to all eligible participants, including details about the purpose, associated risks, potential benefits, and any possible adverse effects. This ensures that participants have a clear and thorough understanding of the relevant information. Only after confirming that participants have been fully informed, they are requested to sign an informed consent form, thus demonstrating their voluntary agreement to participate in the study. The improvement of adherence lies in the need for adequate communication before enrollment, so that the participants completely understand the significance and responsibilities of this study and volunteer to participate.

For peer review only

3.4 Competing interests statement

The authors declare that the research was conducted in the absence of any commercial or financial relationships that could be construed as a potential conflict of interest.

3.5 Access to data

Principal investigators and the study statistician will have access to the final data set. To ensure confidentiality, data dispersed to project team members will be blinded of any identifying participant information.

3.6 Dissemination policy

The findings of this study will be spread through networks of scientists, professionals, and the general public as well as peer-reviewed scientific papers and presentations at pertinent conferences.

4 References

1. Allman C, Amadi U, Winkler AM, et al. Ipsilesional anodal tDCS enhances the functional benefits of rehabilitation in patients after stroke. *Sci Transl Med*. 2016;8(330):330re1. doi:10.1126/scitranslmed.aad5651

2. Hosomi K, Morris S, Sakamoto T, et al. Daily Repetitive Transcranial Magnetic Stimulation for Poststroke Upper Limb Paresis in the Subacute Period. *J Stroke Cerebrovasc Dis*. 2016;25(7):1655-1664. doi:10.1016/j.jstrokecerebrovasdis.2016.02.024

3. Turolla A, Dam M, Ventura L, et al. Virtual reality for the rehabilitation of the upper limb motor function after stroke: a prospective controlled trial. *J Neuroeng Rehabil*. 2013;10:85. doi:10.1186/1743-0003-10-85

4. Persson HC, Parziali M, Danielsson A, Sunnerhagen KS. Outcome and upper extremity function within 72 hours after first occasion of stroke in an unselected population at a stroke unit. A part of the SALGOT study. *BMC Neurol*. 2012;12:162. doi:10.1186/1471-2377-12-162

5. Fisicaro F, Lanza G, Grasso AA, et al. Repetitive transcranial magnetic stimulation in stroke rehabilitation: review of the current evidence and pitfalls. *Ther Adv Neurol Disord*. 2019;12:1756286419878317. doi:10.1177/1756286419878317

6. Murase N, Duque J, Mazzocchio R, Cohen LG. Influence of interhemispheric interactions on motor function in chronic stroke. *Ann Neurol*. 2004;55(3):400-409. doi:10.1002/ana.10848

7. Adeyemo BO, Simis M, Macea DD, Fregni F. Systematic review of parameters of stimulation, clinical trial design characteristics, and motor outcomes in non-invasive brain stimulation in stroke. *Front Psychiatry*. 2012;3:88. doi:10.3389/fpsyt.2012.00088

8. Buma F, Kwakkel G, Ramsey N. Understanding upper limb recovery after stroke. *Restor Neurol Neurosci*. 2013;31(6):707-722. doi:10.3233/RNN-130332

For peer review only

For peer review only - http://bmjopen.bmj.com/site/about/guidelines.xhtml

9. Lefaucheur JP, Aleman A, Baeken C, et al. Evidence-based guidelines on the therapeutic use of repetitive transcranial magnetic stimulation (rTMS): An update (2014-2018). *Clin Neurophysiol*. 2020;131(2):474-528. doi:10.1016/j.clinph.2019.11.002

10. Cunningham DA, Knutson JS, Sankarasubramanian V, Potter-Baker KA, Machado AG, Plow EB. Bilateral Contralaterally Controlled Functional Electrical Stimulation Reveals New Insights Into the Interhemispheric Competition Model in Chronic Stroke. *Neurorehabil Neural Repair*. 2019;33(9):707-717. doi:10.1177/1545968319863709

11. Yang Y, Pan H, Pan W, et al. Repetitive Transcranial Magnetic Stimulation on the Affected Hemisphere Enhances Hand Functional Recovery in Subacute Adult Stroke Patients: A Randomized Trial. *Front Aging Neurosci*. 2021;13:636184. doi:10.3389/fnagi.2021.636184

12. Dodd KC, Nair VA, Prabhakaran V. Role of the Contralesional vs. Ipsilesional Hemisphere in Stroke Recovery. *Front Hum Neurosci*. 2017;11:469. doi:10.3389/fnhum.2017.00469

13. Di Pino G, Pellegrino G, Assenza G, et al. Modulation of brain plasticity in stroke: a novel model for neurorehabilitation. *Nat Rev Neurol*. 2014;10(10):597-608. doi:10.1038/nrneurol.2014.162

14. Plow EB, Sankarasubramanian V, Cunningham DA, et al. Models to Tailor Brain Stimulation Therapies in Stroke. *Neural Plast*. 2016;2016:4071620. doi:10.1155/2016/4071620

15. Sankarasubramanian V, Machado AG, Conforto AB, et al. Inhibition versus facilitation of contralesional motor cortices in stroke: Deriving a model to tailor brain stimulation. *Clin Neurophysiol*. 2017;128(6):892-902. doi:10.1016/j.clinph.2017.03.030

16. Kantak SS, Stinear JW, Buch ER, Cohen LG. Rewiring the brain: potential role of the premotor cortex in motor control, learning, and recovery of function following brain injury. *Neurorehabil Neural Repair*. 2012;26(3):282-292. doi:10.1177/1545968311420845

17. Johansen-Berg H, Rushworth MFS, Bogdanovic MD, Kischka U, Wimalaratna S, Matthews PM. The role of ipsilateral premotor cortex in hand movement after stroke. *Proc Natl Acad Sci U S A*. 2002;99(22):14518-14523. doi:10.1073/pnas.222536799

18. Lotze M, Beutling W, Loibl M, et al. Contralesional motor cortex activation depends on ipsilesional corticospinal tract integrity in well-recovered subcortical stroke patients. *Neurorehabil Neural Repair*. 2012;26(6):594-603. doi:10.1177/1545968311427706

19. Bestmann S, Swayne O, Blankenburg F, et al. The role of contralesional dorsal premotor cortex after stroke as studied with concurrent TMS-fMRI. *J Neurosci*. 2010;30(36):11926-11937. doi:10.1523/JNEUROSCI.5642-09.2010

20. Orihuela-Espina F, Fernández del Castillo I, Palafox L, et al. Neural reorganization accompanying upper limb motor rehabilitation from stroke with virtual reality-based gesture therapy. *Top Stroke Rehabil*. 2013;20(3):197-209. doi:10.1310/tsr2003-197

21. Seniów J, Bilik M, Leśniak M, Waldowski K, Iwański S, Członkowska A. Transcranial magnetic stimulation combined with physiotherapy in rehabilitation of poststroke hemiparesis: a randomized, double-blind, placebo-controlled study. *Neurorehabil Neural Repair*. 2012;26(9):1072-1079. doi:10.1177/1545968312445635

For peer review only

22. Guan YZ, Li J, Zhang XW, et al. Effectiveness of repetitive transcranial magnetic stimulation (rTMS) after acute stroke: A one-year longitudinal randomized trial. *CNS Neurosci Ther*. 2017;23(12):940-946. doi:10.1111/cns.12762

23. Rossini PM, Burke D, Chen R, et al. Non-invasive electrical and magnetic stimulation of the brain, spinal cord, roots and peripheral nerves: Basic principles and procedures for routine clinical and research application. An updated report from an I.F.C.N. Committee. *Clinical Neurophysiology*. 2015;126(6):1071-1107. doi:10.1016/j.clinph.2015.02.001

24. Niérat MC, Similowski T, Lamy JC. Does trans-spinal direct current stimulation alter phrenic motoneurons and respiratory neuromechanical outputs in humans? A double-blind, sham-controlled, randomized, crossover study. *J Neurosci*. 2014;34(43):14420-14429. doi:10.1523/JNEUROSCI.1288-14.2014

25. Duncan PW, Propst M, Nelson SG. Reliability of the Fugl-Meyer assessment of sensorimotor recovery following cerebrovascular accident. *Phys Ther*. 1983;63(10):1606-1610. doi:10.1093/ptj/63.10.1606

26. Lang CE, Bland MD, Bailey RR, Schaefer SY, Birkenmeier RL. Assessment of upper extremity impairment, function, and activity after stroke: foundations for clinical decision making. *J Hand Ther*. 2013;26(2):104-114;quiz 115. doi:10.1016/j.jht.2012.06.005

27. Bai Y, Liu F, Zhang H. Artificial Intelligence Limb Rehabilitation System on Account of Virtual Reality Technology on Long-Term Health Management of Stroke Patients in the Context of the Internet. *Comput Math Methods Med*. 2022;2022:2688003. doi:10.1155/2022/2688003

28. Shah S, Vanclay F, Cooper B. Improving the sensitivity of the Barthel Index for stroke rehabilitation. *J Clin Epidemiol*. 1989;42(8):703-709. doi:10.1016/0895-4356(89)90065-6

29. Meseguer-Henarejos AB, Sánchez-Meca J, López-Pina JA, Carles-Hernández R. Inter- and intra-rater reliability of the Modified Ashworth Scale: a systematic review and meta-analysis. *Eur J Phys Rehabil Med*. 2018;54(4):576-590. doi:10.23736/S1973-9087.17.04796-7

30. Bradnam LV, Stinear CM, Barber PA, Byblow WD. Contralesional hemisphere control of the proximal paretic upper limb following stroke. *Cereb Cortex*. 2012;22(11):2662-2671. doi:10.1093/cercor/bhr344

31. Wilde EA, McCauley SR, Chu Z, et al. Diffusion tensor imaging of hemispheric asymmetries in the developing brain. *J Clin Exp Neuropsychol*. 2009;31(2):205-218. doi:10.1080/13803390802098118

32. Cui Z, Zhong S, Xu P, He Y, Gong G. PANDA: a pipeline toolbox for analyzing brain diffusion images. *Front Hum Neurosci*. 2013;7:42. doi:10.3389/fnhum.2013.00042

33. Rossi S, Hallett M, Rossini PM, Pascual-Leone A, Safety of TMS Consensus Group. Safety, ethical considerations, and application guidelines for the use of transcranial magnetic stimulation in clinical practice and research. *Clin Neurophysiol*. 2009;120(12):2008-2039. doi:10.1016/j.clinph.2009.08.016

For peer review only

5 Author Contributions

TW and YS conceived and designed the study; WD, CL, XY, YZ and LF performed the study and collect materials; HD, CG and YQ analyzed the results; WD, XY and MD wrote the manuscript; YS, TW and WD helped coordinate the study and reviewed the manuscript. All authors contributed to the article and approved the submitted version.

6 Funding statement

This study was funded by the National Key Research &Development Program of China (2022YFC2009700) and the Nanjing Municipal Science and Technology Bureau（2019060002).

7 Patient and Public Involvement

Not applicable. The patient and public will not be involved in the design, conduct, reporting or dissemination of the study.

8 Acknowledgments

We thank all the patients for their contributions during the completion of this study.

Figure 1. Experiment flowchart.

FMA-UE, Fugl-Meyer Assessment of Upper Extremity; AMI, arm sub-score of the Motricity Index; FTHUE-HK, Hong Kong edition of Functional Test for the Hemiplegic Upper Extremity; MBI, Modified Barthel Index; MAS, Modified Ashworth Scale; FA, fractional anisotropy; FAAI, FA asymmetry index; rTMS, repetitive transcranial magnetic stimulation.

Figure 2. Schedule of participant enrollment, interventions, and assessments.

LF-rTMS, low-frequency repetitive transcranial magnetic stimulation; HF-rTMS, high-frequency repetitive transcranial magnetic stimulation; FMA-UE, Fugl-Meyer Assessment of Upper Extremity; AMI, arm sub-score of the Motricity Index; FTHUE-HK, Hong Kong edition of Functional Test for the Hemiplegic Upper Extremity; MBI, Modified Barthel Index; MAS, Modified Ashworth Scale; fMRI, functional magnetic resonance imaging.

For peer review only

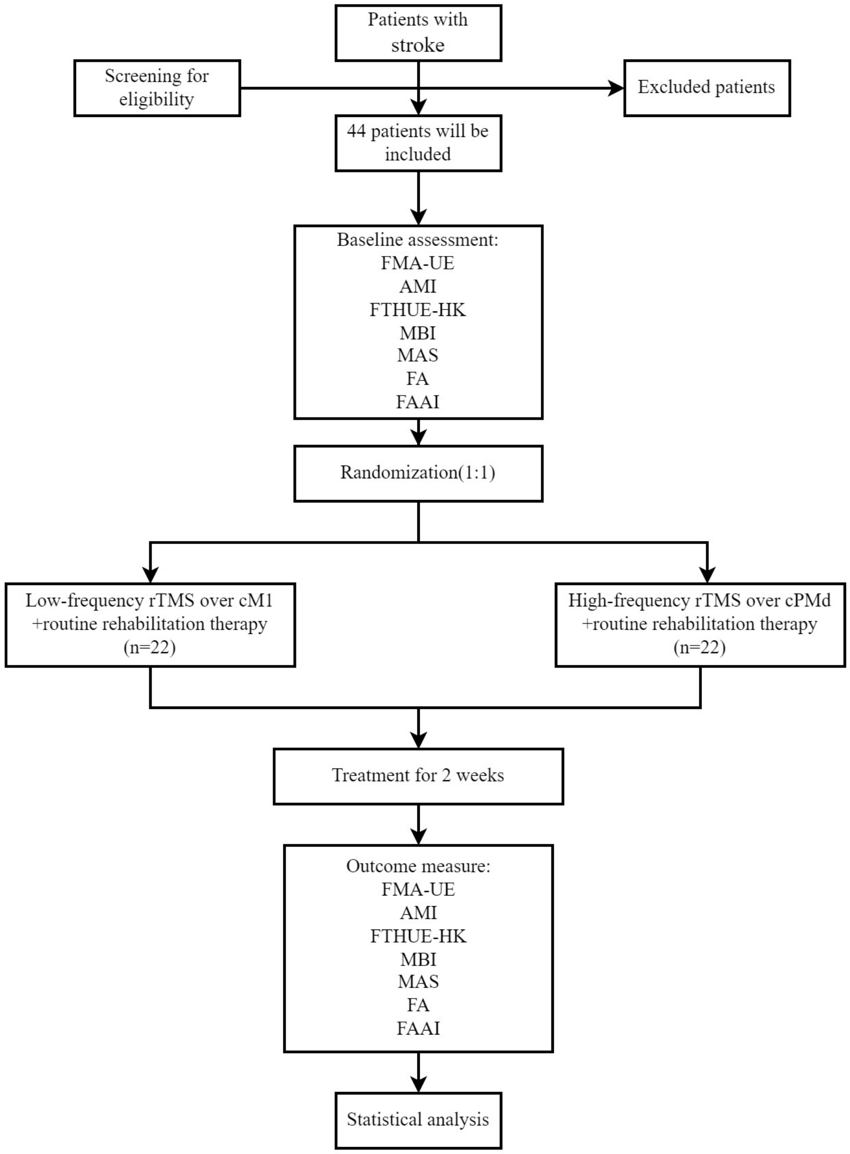

Figure1. Experiment flowchart

460x615mm (72 x 72 DPI)

	Study Period			
	Enrollment	Allocation	Post-allocation	
**TIME POINT**	W-1	0	W1	W2
**ENROLLMENT:**				
Eligibility screen	×			
Informed consent	×			
Randomization	×			
Allocation		×		
**INTERVENTIONS:**				
LF-rTMS protocol over cM1			↔	↔
HF-rTMS protocol over cPMd			↔	↔
**ASSESSMENTS:**				
FMA-UE	×			×
AMI	×			×
FTHUE-HK	×			×
MBI	×			×
MAS	×			×
fMRI	×			×

Figure2.Schedule of participant enrollment, interventions, and assessments

337x259mm (144 x 144 DPI)

SPIRIT

STANDARD PROTOCOL ITEMS: RECOMMENDATIONS FOR INTERVENTIONAL TRIALS

SPIRIT 2013 Checklist: Recommended items to address in a clinical trial protocol and related documents*

Section/item	Item No	Description
**Administrative information**		
Title	1	Descriptive title identifying the study design, population, interventions, and, if applicable, trial acronym ☑ line1-4, page1
Trial registration	2a	Trial identifier and registry name. If not yet registered, name of intended registry ☑ line38, page1
	2b	All items from the World Health Organization Trial Registration Data Set ☑ line415-419, page11
Protocol version	3	Date and version identifier ☑ line282, page7
Funding	4	Sources and types of financial, material, and other support ☑ line405-407, page11
Roles and responsibilities	5a	Names, affiliations, and roles of protocol contributors ☑ line5-12, page1;
	5b	Name and contact information for the trial sponsor ☑ line13-17, page1
	5c	Role of study sponsor and funders, if any, in study design; collection, management, analysis, and interpretation of data; writing of the report; and the decision to submit the report for publication, including whether they will have ultimate authority over any of these activities ☑ line400-404, page10-11
	5d	Composition, roles, and responsibilities of the coordinating centre, steering committee, endpoint adjudication committee, data management team, and other individuals or groups overseeing the trial, if applicable (see Item 21a for data monitoring committee) ☑ line400-404, page10-11
**Introduction**		
Background and rationale	6a	Description of research question and justification for undertaking the trial, including summary of relevant studies (published and unpublished) examining benefits and harms for each intervention ☑ line50-91, page2-3
	6b	Explanation for choice of comparators ☑ line92-100, page3
Objectives	7	Specific objectives or hypotheses ☑ line100-104, page3

For peer review only - http://bmjopen.bmj.com/site/about/guidelines.xhtml

Trial design	8	Description of trial design including type of trial (eg, parallel group, crossover, factorial, single group), allocation ratio, and framework (eg, superiority, equivalence, noninferiority, exploratory) ☑ line106, page3

Methods: Participants, interventions, and outcomes

Study setting	9	Description of study settings (eg, community clinic, academic hospital) and list of countries where data will be collected. Reference to where list of study sites can be obtained ☑ line106-108, page3
Eligibility criteria	10	Inclusion and exclusion criteria for participants. If applicable, eligibility criteria for study centres and individuals who will perform the interventions (eg, surgeons, psychotherapists) ☑ line120-137, page3-4
Interventions	11a	Interventions for each group with sufficient detail to allow replication, including how and when they will be administered ☑ line152-171, page4-5
	11b	Criteria for discontinuing or modifying allocated interventions for a given trial participant (eg, drug dose change in response to harms, participant request, or improving/worsening disease) ☑ line264-271, page7
	11c	Strategies to improve adherence to intervention protocols, and any procedures for monitoring adherence (eg, drug tablet return, laboratory tests) ☑ line288-290, page7
	11d	Relevant concomitant care and interventions that are permitted or prohibited during the trial ☑ line153-157, page4
Outcomes	12	Primary, secondary, and other outcomes, including the specific measurement variable (eg, systolic blood pressure), analysis metric (eg, change from baseline, final value, time to event), method of aggregation (eg, median, proportion), and time point for each outcome. Explanation of the clinical relevance of chosen efficacy and harm outcomes is strongly recommended ☑ line172-212 ,page5
Participant timeline	13	Time schedule of enrolment, interventions (including any run-ins and washouts), assessments, and visits for participants. A schematic diagram is highly recommended (see Figure) ☑ Figure 2 (line419-423, page11)
Sample size	14	Estimated number of participants needed to achieve study objectives and how it was determined, including clinical and statistical assumptions supporting any sample size calculations ☑ line138-144 ,page4
Recruitment	15	Strategies for achieving adequate participant enrolment to reach target sample size ☑ line106-110 ,page3

Methods: Assignment of interventions (for controlled trials)

Allocation:

Sequence generation	16a	Method of generating the allocation sequence (eg, computer-generated random numbers), and list of any factors for stratification. To reduce predictability of a random sequence, details of any planned restriction (eg, blocking) should be provided in a separate document that is unavailable to those who enrol participants or assign interventions ☑ line146-149, page4
Allocation concealment mechanism	16b	Mechanism of implementing the allocation sequence (eg, central telephone; sequentially numbered, opaque, sealed envelopes), describing any steps to conceal the sequence until interventions are assigned ☑ line146-148, page4
Implementation	16c	Who will generate the allocation sequence, who will enrol participants, and who will assign participants to interventions ☑ line149-151, page4
Blinding (masking)	17a	Who will be blinded after assignment to interventions (eg, trial participants, care providers, outcome assessors, data analysts), and how ☑ line149-151, page4
	17b	If blinded, circumstances under which unblinding is permissible, and procedure for revealing a participant's allocated intervention during the trial ☒ only evaluator is blinded

Methods: Data collection, management, and analysis

Data collection methods	18a	Plans for assessment and collection of outcome, baseline, and other trial data, including any related processes to promote data quality (eg, duplicate measurements, training of assessors) and a description of study instruments (eg, questionnaires, laboratory tests) along with their reliability and validity, if known. Reference to where data collection forms can be found, if not in the protocol ☑ line247-249, page6
	18b	Plans to promote participant retention and complete follow-up, including list of any outcome data to be collected for participants who discontinue or deviate from intervention protocols ☑ line248-250, page6
Data management	19	Plans for data entry, coding, security, and storage, including any related processes to promote data quality (eg, double data entry; range checks for data values). Reference to where details of data management procedures can be found, if not in the protocol ☑ line250-252, page6
Statistical methods	20a	Statistical methods for analysing primary and secondary outcomes. Reference to where other details of the statistical analysis plan can be found, if not in the protocol ☑ line213-246, page6

For peer review only - http://bmjopen.bmj.com/site/about/guidelines.xhtml

	20b	Methods for any additional analyses (eg, subgroup and adjusted analyses) ☒ Methods for any additional analyses were not employed in this study.
	20c	Definition of analysis population relating to protocol non-adherence (eg, as randomised analysis), and any statistical methods to handle missing data (eg, multiple imputation) ☑ line225-230, page6
**Methods: Monitoring**		
Data monitoring	21a	Composition of data monitoring committee (DMC); summary of its role and reporting structure; statement of whether it is independent from the sponsor and competing interests; and reference to where further details about its charter can be found, if not in the protocol. Alternatively, an explanation of why a DMC is not needed ☑ line253-262, page7
	21b	Description of any interim analyses and stopping guidelines, including who will have access to these interim results and make the final decision to terminate the trial ☑ line268-271, page7
Harms	22	Plans for collecting, assessing, reporting, and managing solicited and spontaneously reported adverse events and other unintended effects of trial interventions or trial conduct ☑ line263-273, page7
Auditing	23	Frequency and procedures for auditing trial conduct, if any, and whether the process will be independent from investigators and the sponsor ☑ line271-273, page7
**Ethics and dissemination**		
Research ethics approval	24	Plans for seeking research ethics committee/institutional review board (REC/IRB) approval ☑ line275-277, page7
Protocol amendments	25	Plans for communicating important protocol modifications (eg, changes to eligibility criteria, outcomes, analyses) to relevant parties (eg, investigators, REC/IRBs, trial participants, trial registries, journals, regulators) ☑ line278- 282, page7
Consent or assent	26a	Who will obtain informed consent or assent from potential trial participants or authorised surrogates, and how (see Item 32) ☑ line283-290, page7
	26b	Additional consent provisions for collection and use of participant data and biological specimens in ancillary studies, if applicable ☒This content wasn't involved in the study.
Confidentiality	27	How personal information about potential and enrolled participants will be collected, shared, and maintained in order to protect confidentiality before, during, and after the trial ☑ line250-252, page6
Declaration of interests	28	Financial and other competing interests for principal investigators for the overall trial and each study site ☑ line291-293, page7-8

eview only

Access to data	29	Statement of who will have access to the final trial dataset, and disclosure of contractual agreements that limit such access for investigators ☑ line294-297, page8
Ancillary and post-trial care	30	Provisions, if any, for ancillary and post-trial care, and for compensation to those who suffer harm from trial participation ☒ N/A
Dissemination policy	31a	Plans for investigators and sponsor to communicate trial results to participants, healthcare professionals, the public, and other relevant groups (eg, via publication, reporting in results databases, or other data sharing arrangements), including any publication restrictions ☑ line298-300, page8
	31b	Authorship eligibility guidelines and any intended use of professional writers ☑ line298-300, page8
	31c	Plans, if any, for granting public access to the full protocol, participant-level dataset, and statistical code ☒ There weren't plans for granting public access to the full protocol, participant-level dataset, and statistical code.
**Appendices**		
Informed consent materials	32	Model consent form and other related documentation given to participants and authorised surrogates ☑ the patient consent form in the supplementary file
Biological specimens	33	Plans for collection, laboratory evaluation, and storage of biological specimens for genetic or molecular analysis in the current trial and for future use in ancillary studies, if applicable ☒ There weren't plans for collection, laboratory evaluation, or storage of biological specimens in this study.

*It is strongly recommended that this checklist be read in conjunction with the SPIRIT 2013 Explanation & Elaboration for important clarification on the items. Amendments to the protocol should be tracked and dated. The SPIRIT checklist is copyrighted by the SPIRIT Group under the Creative Commons "Attribution-NonCommercial-NoDerivs 3.0 Unported" license.

For peer review only - http://bmjopen.bmj.com/site/about/guidelines.xhtml

BMJ Open

Effects of repetitive transcranial magnetic stimulation over the contralesional dorsal premotor cortex on upper limb function in severe ischemic stroke: study protocol for a randomized controlled trial

Journal:	*BMJ Open*
Manuscript ID	bmjopen-2023-074037.R2
Article Type:	Protocol
Date Submitted by the Author:	07-Oct-2023
Complete List of Authors:	Dai, Wenjun; Jiangsu Province Hospital and Nanjing Medical University First Affiliated Hospital, Rehabilitation Medicine Center<br>Yang, Xi; Nanjing Medical University, School of Rehabilitation Medicine<br>Liu, Canhuan; Nanjing Medical University, School of Rehabilitation Medicine<br>Ding, Hongyuan; Jiangsu Province Hospital and Nanjing Medical University First Affiliated Hospital, Department of Radiology<br>Guo, Chuan; Jiangsu Province Hospital and Nanjing Medical University First Affiliated Hospital, Rehabilitation Medicine Center<br>Zhu, Yi; Jiangsu Province Hospital and Nanjing Medical University First Affiliated Hospital, Rehabilitation Medicine Center<br>Dong, Manyu; Nanjing Medical University, School of Rehabilitation Medicine<br>Qian, Yilun; Nanjing Medical University, School of Rehabilitation Medicine<br>Fang, Lu; Jiangsu Province Hospital and Nanjing Medical University First Affiliated Hospital, Rehabilitation Medicine Center<br>Wang, Tong; Jiangsu Province Hospital and Nanjing Medical University First Affiliated Hospital, Rehabilitation Medicine Center<br>Shen, Ying; Jiangsu Province Hospital and Nanjing Medical University First Affiliated Hospital, Rehabilitation Medicine Center
**Primary Subject Heading**:	Rehabilitation medicine
Secondary Subject Heading:	Neurology
Keywords:	Stroke < NEUROLOGY, REHABILITATION MEDICINE, Clinical trials < THERAPEUTICS

SCHOLARONE™
Manuscripts

Effects of repetitive transcranial magnetic stimulation over the contralesional dorsal premotor cortex on upper limb function in severe ischemic stroke: study protocol for a randomized controlled trial

Wenjun Dai[1#], Xi Yang[2#], Canhuan Liu[2#], Hongyuan Ding[3#], Chuan Guo[1], Yi Zhu[1], Manyu Dong[2], Yilun Qian[2], Lu Fang[1], Tong Wang[1*], Ying Shen[1*]

[1] Rehabilitation Medicine Center, The First Affiliated Hospital of Nanjing Medical University, Nanjing, China

[2] School of Rehabilitation Medicine, Nanjing Medical University, Nanjing, China

[3] Department of Radiology, The First Affiliated Hospital of Nanjing Medical University, Nanjing, China

[#]These authors have contributed equally to this work and share first authorship

*** Correspondence:**

Ying Shen

shenying@njmu.edu.cn

Tong Wang

wangtong60621@163.com

For peer review only

Abstract

Introduction: Repetitive transcranial magnetic stimulation (rTMS) is an evidence-based treatment widely recommended to promote hand motor recovery after ischemic stroke. However, the therapeutic efficacy of rTMS over the motor cortex in stroke patients is currently restricted and heterogeneous. This study aimed to determine whether excitatory rTMS over the contralesional dorsal premotor cortex (cPMd) facilitates the functional recovery of the upper limbs during the post-acute stage of severe ischemic stroke.

Methods and Analysis: This study will be conducted as a single-blind, controlled, randomized study, in which 44 patients with poststroke hemiplegia with a course of disease ranging from 1 week

to 3 months and Fugl-Meyer upper limb score ≤22 will be enrolled. The study participants will be randomly assigned to groups A (n=22) and B (n=22). The two groups are based on routine rehabilitation training and drug treatment; group A will be treated with low-frequency (1 Hz) rTMS over the contralesional primary motor cortex (cM1), and group B will be treated with high-frequency rTMS (10 Hz) over cPMd. For 2 weeks, rTMS will be administered once a day, 5 days a week. The primary outcome is the Fugl–Meyer assessment of the upper limb. The secondary outcomes include the arm sub-score of the motricity index (AMI), Hong Kong edition of Functional Test for the Hemiplegic Upper Extremity (FTHUE-HK), Modified Barthel Index (MBI), and Modified Ashworth Scale (MAS) score of the paralyzed pectoralis major (PM) and biceps brachii (BI). Furthermore, data of diffusion tensor imaging (DTI) and functional magnetic resonance imaging (fMRI) will be collected. These outcomes will be assessed before and after the completion of the intervention.

Ethics and dissemination

This study has been approved by the Ethics Committee of First Affiliated Hospital of Nanjing Medical University(2020-SR-266) . The findings of this study will be spread through networks of scientists, professionals, and the general public as well as peer-reviewed scientific papers and presentations at pertinent conferences.

Clinical Trial registration number Chinese Clinical Trial Registry ChiCTR2000038049

Strengths and limitations of this study

1. This will be a single-blinded, controlled, randomized clinical study to compare high-frequency rTMS over cPMd and low-frequency rTMS over cM1 in severe ischemic stroke.
2. We will use neuronavigation to position the site of cPMd and cM1 stimulation.
3. Additionally, various outcome measures, including clinical function and functional magnetic resonance imaging, will be included to investigate the therapeutic effects of rTMS and its underlying neuroplasticity mechanisms.
4. Because of low-frequency rTMS to the cM1 ranked as an A-level recommendation for improving motor function after stroke, no blank control group undergoing sham TMS will be set up.
5. Given the nature of TMS interventions, blinding operators is not feasible.

1 Introduction

Ischemic stroke has emerged as a critical public health problem as the main contributor to significant long-term impairment.[1,2] Among them, the age group of 40-70 years old is the primary high-risk age range for stroke, which has relative homogeneity, stability, and representativeness.[3] Movement disorders after ischemic stroke not only reduce the activities of daily living (ADL) and affect the health of patients but also place a heavy strain on the society as a whole.[2,4] Hypofunction of the upper limbs is one of the most common types of dyskinesia following ischemic stroke, which seriously affects the patient's ADL and hampers rehabilitation progression.[5,6] In ischemic stroke rehabilitation, improving the upper extremity functional capacity remains a challenging issue.

The effectiveness of repetitive transcranial magnetic stimulation (rTMS) as ischemic stroke rehabilitation therapy has been thoroughly studied.[6,7] This technique promotes brain plasticity by providing continuous magnetic pulses to the brain .[8] After stroke, neuroplasticity-induced cortical reconfiguration is necessary for the restoration of motor performance.[9] According to widespread consensus, low-frequency (≤1 Hz) rTMS can reduce neuronal activity and cortical excitability, whereas high-frequency (≥5 Hz) rTMS has the opposite effect.[10] The interhemispheric inhibition

For peer review only - http://bmjopen.bmj.com/site/about/guidelines.xhtml

(IHI) model is the foundation of the current rTMS intervention method to support the improvement of upper extremity performance in stroke patients.[11] It indicates that decreased excitability of the lesioned primary motor cortex (M1) region after stroke results from "double obstacles," including the ipsilateral areas and excessive inhibition in the contralateral areas.[12] Therefore, lowering interhemispheric inhibition by minimizing the excitation of the contralesional side will improve recovery.[13] The IHI model currently serves as the foundation for recommendations regarding the application of rTMS in stroke recovery, with low-frequency rTMS to the contralesional M1 (cM1) ranked as an A-level recommendation for improving motor function.[10] Because the heterogeneity of patients is not taken into account, the clinical efficacy of this rTMS intervention strategy for poststroke motor dysfunction is limited.[10] However, for patients with severe stroke with low structural retention, using the vicariation model may be preferred, which posits that activity in the remaining network locations compensates for the loss of certain capabilities in damaged areas.[14]

In patients with severe ischemic stroke, the dorsal premotor cortex (PMd) of the contralesional hemisphere plays an impressive compensatory role in motor function recovery.[15,16] PMd has extensive connections to M1 as well as specific motor regions in the cerebral motor connections of the parietal and frontal lobes.[17] Vishwanath et al. found that the novel approach of cPMd facilitation (5Hz rTMS) resulted in more improvement than cM1 inhibition (1Hz rTMS) in severely impaired stroke patients by alleviating interhemispheric competition inflicted on weak iM1.[16] cPMd reduces inhibition of weak iM1 and provides ipsilateral access (uncrossed corticospinal and brainstem-mediated reticulospinal) to the paralyzed limb to aid recovery.[15,18] Lotze proposed an inverse correlation between dorsal premotor cortex activation and ipsilateral corticospinal tract integrity, verifying the potential importance of contralateral dorsal premotor cortex for good motor performance.[19] In a study on interhemispheric cPMd-iM1 interactions, Bestmann et al. used paired-coil TMS through concurrent TMS-fMRI and confirmed that conditioning cPMd pulses had a facilitative effect on ipsilesional M1.[20] This depends on the rising excitability in ipsilesional sensorimotor areas (BA4p), which maintains direct projections to spinal motoneurons and then increases the motor outputs of the paralyzed upper limb.[20] Therefore, we expect that cPMd, as a potential therapeutic target, can facilitate the rehabilitation of upper extremity movements following a ischemic stroke.

To this end, this randomized controlled clinical trial aims to investigate whether the excitatory rTMS protocol over cPMd has a better effect than the inhibitory rTMS protocol over cM1 to compensate for the limitations of its inconsistent therapeutic effects. In addition to common clinical scales for evaluating upper limb motor function, diffusion tensor imaging (DTI) and functional magnetic resonance imaging (fMRI) will be used to explore changes in structural and functional connectivity, analyze the correlation between clinical function assessments and neuroimaging data, and further elucidate the neurological mechanisms underlying poststroke upper limb rehabilitation.[21] Several previous investigations have shown that cM1 inhibition does not significantly increase the rehabilitation of upper extremity movements in severe stroke patients.[16,22] As a result, we hypothesize that the excitatory rTMS protocol over cPMd might be an effective therapy for the recovery of upper extremity motor performance following severe ischemic stroke. This research may offer significant novel clues into the clinical effects of rTMS in the treatment of upper extremity impairment in ischemic stroke patients and the underlying neuroplasticity mechanisms of TMS intervention.

2 Materials and Analysis

For peer review only

This study is a single-blind, controlled, randomized clinical trial with two groups (A and B). Study will be carried out in the Rehabilitation Medicine Center, The First Affiliated Hospital of Nanjing Medical University, Nanjing, China. Before the trial, detailed information about the research project was provided to potential participants. After the patients voluntarily sign the informed consent form, they will be invited to participate in the trial. The improvement of adherence lies in the need for adequate communication before enrollment, so that the participants completely understand the significance and responsibilities of this study and volunteer to participate.

All eligible participants will be randomly assigned (1:1) to either group A (n=22) or group B (n=22). In consideration of research purposes, ethical perspectives, feasibility, and practicality, etc., this study was not designed with a placebo control group. **Figure 1** depicts the experiment flowchart. The study protocol has been approved by the Ethics Committee of the First Affiliated Hospital of Nanjing Medical University (2020-SR-266) and registered on Chinese Clinical Trial Registry (ChiCTR2000038049, website: http:// www.chictr.org.cn).

2.1 Participants

2.1.1 Inclusion criteria

The following are the inclusion criteria:(1) ischemic stroke confirmed by computed tomography (CT) and/or MRI meets the diagnostic criteria for cerebral infarction in the Cerebrovascular Disease; (2) primary or unilateral onset or previous onset without residual neurological dysfunction; (3) stable vital signs and clear consciousness; (4) the age ranges from 40 to 70 years; (5) the course of disease ranges from 1 week to 3 months; (6) paralysis of the upper limbs; (7) FMA-UE score ≤ 22; and (8) the selected candidate or his relatives sign the informed consent form.

2.1.2 Exclusion criteria

The following are the exclusion criteria: (1) epilepsy in the previous, a family history of epilepsy, and taking medications that cause seizures; (2) the function of the urinary, cardiovascular, respiratory, and other important systems decreases or fails; (3) severe deficits in cognition and communication that impede patient participation during evaluation and therapy; (4) posterior circulation infarction; (5) TMS and fMRI-related restrictions, including a pacemaker, an artificial metal heart valve implant, a drug treatment pump, an insulin pump, an aneurysm clip (other than titanium alloy, which is not paramagnetic), and a metal implant in the body; (6) severe cervical spondylosis includes severe cervical stenosis and cervical spinal instability; (7) complete occlusion of the internal carotid artery; (8) direct injuries and skull defects in the stimulation area; (9) women during pregnancy; (10) severe high fever; (11) claustrophobia; and (12) inability to cooperate with fMRI examination.

2.2 Sample size

The sample size was estimated using GPower (version 3.1.9.7). To evaluate the impact of LF-rTMS protocol over cM1 versus HF-rTMS protocol over cPMd on functional motor performance of the upper extremities evaluated utilizing FMA-UE (the primary outcome) over 2 weeks, a sample size of 42 patients (21 per group) completing the intervention will be expected to reach the statistical power of 80% at the significance level of 0.05 (two-sided test), assuming a small-to-moderate effect size of Cohen's d=0.45.[23] We will randomize 44 patients (22 in each group) to account for a 5% dropout rate.

2.3 Randomization and blinding

For peer review only

After the baseline assessment is completed and the inclusion/exclusion criteria are checked, the participants will be randomized into group A or B (ratio 1:1) using SPSS software to generate random sequences. The stratification criterion is the course duration of the disease, with two strata: 1 month and 2-3 months. This study is a single-blind study. However, the evaluators who perform the measurements will be blinded to the group to which the participants belong. Special personnel are responsible for randomization, assessment and intervention, respectively.

2.4 Intervention

All patients will receive current routine pharmacological treatments and an 80-min rehabilitation therapy session that combines physical therapy for upper limb function, neuromuscular electrical stimulation, occupational therapy, and rTMS therapy. Over the course of 2 weeks, all treatments will be administered once daily, 5 days a week (10 sessions in total). The relevant period segments of the experiment are shown in **Figure 2**.

rTMS will be applied with a CCY-I TMS stimulator (Yiruide Co., Wuhan, China) using a figure-8-shaped coil for accurate targeted treatment. The center of the coil will be tangential to the treatment plane, and the handle will be positioned at 45° from the sagittal plane. Using the surface electromyography (EMG) data that will be collected from the abductor pollicis brevis on the uninjured side, the potential difference between the highest and lowest peaks will be selected to obtain the motor-evoked potential (MEP). The minimal intensity required to generate at least 5 out of 10 MEPs in a relaxed target muscle that are >50 μV is known as the resting motor threshold (RMT).[24] The group A strategy will use rTMS (1 Hz) over cM1, 90% RMT, 120 trains of 10-s duration, 2-s inter-trial intervals, 1200 pulses per session, and a 23-min, 58-s total duration. The group B strategy will use rTMS (10 Hz) over cPMd, 90% RMT, 80 trains of 1.5-s duration, 10-s inter-trial intervals, 1200 pulses per session, and a 15-min, 10-s total duration. The coil positioning will be guided throughout the experiment using a neuronavigation system (Visor2 ANT; Germany). [25] For a period of 2 weeks, each patient will receive rTMS stimulation once each day (5 days each week).

2.5 Outcome Assessment

2.5.1 Primary Outcome

FMA-UE will be used to evaluate performance-based motor functions of the paralyzed upper limbs. The FMA-UE consists of 9 major items and 33 sub-items. Each item receives a rating between 0 and 3, and the total score ranges from 0 (no movement) to 66 (normal active movement). A higher score indicates lower motor impairment.[26]

2.5.2 Secondary Outcomes

2.5.2.1 Clinical and functional assessments

AMI includes shoulder abduction, elbow flexion, and pinch grip strength. The overall score of the scale is calculated by summing the scores of the three components (the highest score for each item is 33 points) and 1 point, so that a total of 100 points is reached. The score is positively correlated with the motor performance of the upper limb.[27]

FTHUE-HK includes 12 test items with 7 functional levels that evaluate upper limb function as a whole. The condition in which the patient has completed the test for this level is determined according to the requirements of each level.[28] A higher level indicates that the patients have better

For peer review only - http://bmjopen.bmj.com/site/about/guidelines.xhtml

upper extremity motor function and a greater capacity for using the upper limbs during daily activities.

MBI[29] is a 10-item rating scale that measures the quality of general life activities. The higher the score, the more independent the patients are in their daily lives. Scores > 60 indicate a high likelihood of performing ADLs and maintaining a standard of living.

MAS is the most widely used clinical scale to assess the increase in muscle tone after central nervous system lesions, which is manifested by increased resistance of joints to passive movement. According to the MAS, possible scores range from 0, 1, 1^+, 2, 3, and 4, with 0 reflecting normal tone and 4 reflecting fixed muscle contracture.[30]

2.5.2.2 DTI and fMRI indicators

MR scanning will be performed using a 3.0T MR scanner (Discovery750W, GE Healthcare, USA) and a 24-channel head coil. In addition to T2WI scans to exclude parenchymal brain lesions and abnormalities, anatomical images will be obtained using a 3D gradient-echo pulse sequence to collect T1–weighted images in the sagittal plane. The following will be the parameters: repetition time (TR)=8.5 ms, time echo (TE)=2.52 ms, inversion time (TI)=450 ms, field of view (FOV)=256×256 mm², matrix size=256×256, flip angle=12°, image matrix=128×128, number of excitations (NEX)=1, bandwidth=31.25 kHz, 1.0-mm thick slices, and 192 slices. Data Processing Assistant Resting-State fMRI (DPARSF) software (http://rfmri.org/DPARSF) will be used to preprocess the functional imaging data. DTI determines lesion size and fractional anisotropy (FA) within the posterior limb of the internal capsule (PLIC) to assess the integrity of the corticospinal tract (CST).[31] DTI is based on an echo-planar imaging (EPI) sequence. The parameters were as follows: TR=8000 ms, TE=96 ms, image matrix=112×112, FOV=22.4×22.4 cm², b=0 and 1000 s/mm², NEX=2, slice thickness=3 mm, interslice spacing=0 mm, and acceleration factor=2. FA values and the mean FA asymmetry index (FAAI) are the leading indicators of tract integrity. FAAI=(FAunaffected−FAaffected)/(FAunaffected+FAaffected)[32]. FAAI is in the range of −1.0 to +1.0.

2.6 Statistical Analysis

2.6.1 Clinical Data Analysis

Statistical analysis will be employed using SPSS statistical software (version 22.0, Chicago, IL, United States). Prior to entering data for descriptive statistics, the Shapiro-Wilk test will be used to verify normal probability. Data from the normal distribution will be given as mean and standard deviation, but data from the non-normal distribution will be expressed as medians with interquartile ranges. Categorical variables will be described as a function of frequency as a percentage. Demographic characteristics and baseline variables will be assessed for between-group differences using statistical tests appropriate for the data type. Specifically, independent sample t-tests or nonparametric Mann-Whitney tests will be used for continuous variables, while chi-square or Fisher's exact tests will be employed for categorical variables. Repeated-measures analysis of variance (ANOVA) will be used to estimate the therapeutic effect for primary (e.g., FMA-UE) and secondary outcomes (e.g., list all secondary outcomes here). Statistical significance is defined as $p<0.05$. The primary analysis will employ an intention-to-treat method to address non-adherence. The absence of primary outcome data will be addressed by utilizing baseline data as a reference point and conducting additional sensitivity analyses. The utilization of a randomized design precludes the use of multivariable models for confounding adjustment. Consequently, the absence of covariates will have no impact on the primary analysis.

For peer review only

2.6.2 Imaging Data Analysis

DTI datasets will be processed using the PANDA toolkit (http://www.nitrc.org/projects/panda) to obtain the average FA and FAAI values of patients from the whole group.[33] The PANDA process involves three major steps: pre-processing, producing diffusion metrics, and constructing networks. The bilateral PLIC, pons, and precentral gyrus are selected as regions of interest (ROIs). Comparisons within groups will be performed using paired t-tests, and those between groups using two-sample t-tests. Structural and functional connectivity will be processed using the Infinitome software (Omniscient Neurotechnology (2020) Infinitome). The statistical significance level is set at $p<0.05$.

For the Human Connectome Project Multimodal Parcellation maps, specific topic versions will be produced using a machine-learning approach. Depending on the paired functioning connection among each participant's brain maps, it will be used to model their diagnostic classification and neuropsychological examination results. A “boosted trees” method will be applied to every case by the XGB Classifier to adapt the model. This strategy offers better predictive capabilities than using a single tree.

2.7 Data collection, management and monitoring

Data collection will be conducted by highly skilled attending physicians, therapists, and radiologists who are independent of the grouping and treatment procedures. They will promptly input the gathered data into the designated case report form (CRF). Subsequently, a designated individual will digitally capture all the data while ensuring the use of unique identifiers to safeguard patient privacy and data security.

The data monitoring committee (DMC) will verify whether the conduct, generation, recording, and reporting of the clinical trial comply with the regulatory requirements of the protocol, based on the monitoring plan and written standard operating procedures. By regularly reviewing the collected data, including checking the data entry and storage against predefined standards and guidelines, they examine the consistency, logic, and validity of the data. They compare the entered data with the source data to confirm its accuracy and consistency. If any anomalies or potential issues are identified, the DMC will promptly investigate and correct them. They may communicate with data collectors, researchers, or other relevant parties to clarify and resolve issues, ensuring the validity and reliability of the data. The goal of data monitoring is to minimize data errors and biases, and ensure data quality to support accurate data analysis and reliable research conclusions.

2.8 Harms

The intensity, frequency, and other parameter settings of rTMS will comply with the safety guidelines[34] issued by the *International Transcranial Magnetic Association* in 2009. To reduce the probability of an adverse event, the participants will be rigorously screened in accordance with the exclusion and inclusion criteria. Adverse effects and events will be closely monitored during the clinical trial. If this occurs, it will be noted in the CRF. Some incidents will be monitored, and when these incidents are inappropriate, the treatment will be discontinued, such as exacerbation of the condition, serious adverse events, poor adherence leading to a loss of follow-up, or the development of a new serious illness affecting the course of this protocol. This study is performed under the

For peer review only

supervision of the Hospital Research Ethics Committee. Study progress and existing problems are semi-annually reported, and adjustments are made to overcome them in a timely manner.

3 Ethics and dissemination

3.1 Research ethics approval

This study has been approved by the Ethics Committee of First Affiliated Hospital of Nanjing Medical University(2020-SR-266) .

3.2 Protocol amendments

The modifications to the protocol will be determined through in-depth discussions and careful deliberations by the Hospital Research Ethics Committee. Once the amendments receive approval from the Chinese Clinical Trial Registry, the modified protocol will be implemented for the study. The current version of the protocol is V5.0 (date: June 28, 2021).

3.3 Consent or assent

The researchers diligently provide comprehensive information to all eligible participants, including details about the purpose, associated risks, potential benefits, and any possible adverse effects. This ensures that participants have a clear and thorough understanding of the relevant information. Only after confirming that participants have been fully informed, they are requested to sign an informed consent form, thus demonstrating their voluntary agreement to participate in the study. The improvement of adherence lies in the need for adequate communication before enrollment, so that the participants completely understand the significance and responsibilities of this study and volunteer to participate.

3.4 Competing interests statement

The authors declare that the research was conducted in the absence of any commercial or financial relationships that could be construed as a potential conflict of interest.

3.5 Access to data

Principal investigators and the study statistician will have access to the final data set. To ensure confidentiality, data dispersed to project team members will be blinded of any identifying participant information.

3.6 Dissemination policy

The findings of this study will be spread through networks of scientists, professionals, and the general public as well as peer-reviewed scientific papers and presentations at pertinent conferences.

4 References

1. Allman C, Amadi U, Winkler AM, et al. Ipsilesional anodal tDCS enhances the functional benefits of rehabilitation in patients after stroke. *Sci Transl Med*. 2016;8(330):330re1. doi:10.1126/scitranslmed.aad5651

For peer review only

2. Hosomi K, Morris S, Sakamoto T, et al. Daily Repetitive Transcranial Magnetic Stimulation for Poststroke Upper Limb Paresis in the Subacute Period. *J Stroke Cerebrovasc Dis*. 2016;25(7):1655-1664. doi:10.1016/j.jstrokecerebrovasdis.2016.02.024

3. Peng JW, Liu Y, Meng G, Zhang JY, Yu LF. Effects of salvianolic acid on cerebral perfusion in patients after acute stroke: A single-center randomized controlled trial. *Exp Ther Med*. 2018;16(3):2600-2614. doi:10.3892/etm.2018.6444

4. Turolla A, Dam M, Ventura L, et al. Virtual reality for the rehabilitation of the upper limb motor function after stroke: a prospective controlled trial. *J Neuroeng Rehabil*. 2013;10:85. doi:10.1186/1743-0003-10-85

5. Persson HC, Parziali M, Danielsson A, Sunnerhagen KS. Outcome and upper extremity function within 72 hours after first occasion of stroke in an unselected population at a stroke unit. A part of the SALGOT study. *BMC Neurol*. 2012;12:162. doi:10.1186/1471-2377-12-162

6. Fisicaro F, Lanza G, Grasso AA, et al. Repetitive transcranial magnetic stimulation in stroke rehabilitation: review of the current evidence and pitfalls. *Ther Adv Neurol Disord*. 2019;12:1756286419878317. doi:10.1177/1756286419878317

7. Murase N, Duque J, Mazzocchio R, Cohen LG. Influence of interhemispheric interactions on motor function in chronic stroke. *Ann Neurol*. 2004;55(3):400-409. doi:10.1002/ana.10848

8. Adeyemo BO, Simis M, Macea DD, Fregni F. Systematic review of parameters of stimulation, clinical trial design characteristics, and motor outcomes in non-invasive brain stimulation in stroke. *Front Psychiatry*. 2012;3:88. doi:10.3389/fpsyt.2012.00088

9. Buma F, Kwakkel G, Ramsey N. Understanding upper limb recovery after stroke. *Restor Neurol Neurosci*. 2013;31(6):707-722. doi:10.3233/RNN-130332

10. Lefaucheur JP, Aleman A, Baeken C, et al. Evidence-based guidelines on the therapeutic use of repetitive transcranial magnetic stimulation (rTMS): An update (2014-2018). *Clin Neurophysiol*. 2020;131(2):474-528. doi:10.1016/j.clinph.2019.11.002

11. Cunningham DA, Knutson JS, Sankarasubramanian V, Potter-Baker KA, Machado AG, Plow EB. Bilateral Contralaterally Controlled Functional Electrical Stimulation Reveals New Insights Into the Interhemispheric Competition Model in Chronic Stroke. *Neurorehabil Neural Repair*. 2019;33(9):707-717. doi:10.1177/1545968319863709

12. Yang Y, Pan H, Pan W, et al. Repetitive Transcranial Magnetic Stimulation on the Affected Hemisphere Enhances Hand Functional Recovery in Subacute Adult Stroke Patients: A Randomized Trial. *Front Aging Neurosci*. 2021;13:636184. doi:10.3389/fnagi.2021.636184

13. Dodd KC, Nair VA, Prabhakaran V. Role of the Contralesional vs. Ipsilesional Hemisphere in Stroke Recovery. *Front Hum Neurosci*. 2017;11:469. doi:10.3389/fnhum.2017.00469

14. Di Pino G, Pellegrino G, Assenza G, et al. Modulation of brain plasticity in stroke: a novel model for neurorehabilitation. *Nat Rev Neurol*. 2014;10(10):597-608. doi:10.1038/nrneurol.2014.162

For peer review only - http://bmjopen.bmj.com/site/about/guidelines.xhtml

15. Plow EB, Sankarasubramanian V, Cunningham DA, et al. Models to Tailor Brain Stimulation Therapies in Stroke. *Neural Plast*. 2016;2016:4071620. doi:10.1155/2016/4071620

16. Sankarasubramanian V, Machado AG, Conforto AB, et al. Inhibition versus facilitation of contralesional motor cortices in stroke: Deriving a model to tailor brain stimulation. *Clin Neurophysiol*. 2017;128(6):892-902. doi:10.1016/j.clinph.2017.03.030

17. Kantak SS, Stinear JW, Buch ER, Cohen LG. Rewiring the brain: potential role of the premotor cortex in motor control, learning, and recovery of function following brain injury. *Neurorehabil Neural Repair*. 2012;26(3):282-292. doi:10.1177/1545968311420845

18. Johansen-Berg H, Rushworth MFS, Bogdanovic MD, Kischka U, Wimalaratna S, Matthews PM. The role of ipsilateral premotor cortex in hand movement after stroke. *Proc Natl Acad Sci U S A*. 2002;99(22):14518-14523. doi:10.1073/pnas.222536799

19. Lotze M, Beutling W, Loibl M, et al. Contralesional motor cortex activation depends on ipsilesional corticospinal tract integrity in well-recovered subcortical stroke patients. *Neurorehabil Neural Repair*. 2012;26(6):594-603. doi:10.1177/1545968311427706

20. Bestmann S, Swayne O, Blankenburg F, et al. The role of contralesional dorsal premotor cortex after stroke as studied with concurrent TMS-fMRI. *J Neurosci*. 2010;30(36):11926-11937. doi:10.1523/JNEUROSCI.5642-09.2010

21. Orihuela-Espina F, Fernández del Castillo I, Palafox L, et al. Neural reorganization accompanying upper limb motor rehabilitation from stroke with virtual reality-based gesture therapy. *Top Stroke Rehabil*. 2013;20(3):197-209. doi:10.1310/tsr2003-197

22. Seniów J, Bilik M, Leśniak M, Waldowski K, Iwański S, Członkowska A. Transcranial magnetic stimulation combined with physiotherapy in rehabilitation of poststroke hemiparesis: a randomized, double-blind, placebo-controlled study. *Neurorehabil Neural Repair*. 2012;26(9):1072-1079. doi:10.1177/1545968312445635

23. Guan YZ, Li J, Zhang XW, et al. Effectiveness of repetitive transcranial magnetic stimulation (rTMS) after acute stroke: A one-year longitudinal randomized trial. *CNS Neurosci Ther*. 2017;23(12):940-946. doi:10.1111/cns.12762

24. Rossini PM, Burke D, Chen R, et al. Non-invasive electrical and magnetic stimulation of the brain, spinal cord, roots and peripheral nerves: Basic principles and procedures for routine clinical and research application. An updated report from an I.F.C.N. Committee. *Clinical Neurophysiology*. 2015;126(6):1071-1107. doi:10.1016/j.clinph.2015.02.001

25. Niérat MC, Similowski T, Lamy JC. Does trans-spinal direct current stimulation alter phrenic motoneurons and respiratory neuromechanical outputs in humans? A double-blind, sham-controlled, randomized, crossover study. *J Neurosci*. 2014;34(43):14420-14429. doi:10.1523/JNEUROSCI.1288-14.2014

26. Duncan PW, Propst M, Nelson SG. Reliability of the Fugl-Meyer assessment of sensorimotor recovery following cerebrovascular accident. *Phys Ther*. 1983;63(10):1606-1610. doi:10.1093/ptj/63.10.1606

For peer review only

27. Lang CE, Bland MD, Bailey RR, Schaefer SY, Birkenmeier RL. Assessment of upper extremity impairment, function, and activity after stroke: foundations for clinical decision making. *J Hand Ther*. 2013;26(2):104-114;quiz 115. doi:10.1016/j.jht.2012.06.005

28. Bai Y, Liu F, Zhang H. Artificial Intelligence Limb Rehabilitation System on Account of Virtual Reality Technology on Long-Term Health Management of Stroke Patients in the Context of the Internet. *Comput Math Methods Med*. 2022;2022:2688003. doi:10.1155/2022/2688003

29. Shah S, Vanclay F, Cooper B. Improving the sensitivity of the Barthel Index for stroke rehabilitation. *J Clin Epidemiol*. 1989;42(8):703-709. doi:10.1016/0895-4356(89)90065-6

30. Meseguer-Henarejos AB, Sánchez-Meca J, López-Pina JA, Carles-Hernández R. Inter- and intra-rater reliability of the Modified Ashworth Scale: a systematic review and meta-analysis. *Eur J Phys Rehabil Med*. 2018;54(4):576-590. doi:10.23736/S1973-9087.17.04796-7

31. Bradnam LV, Stinear CM, Barber PA, Byblow WD. Contralesional hemisphere control of the proximal paretic upper limb following stroke. *Cereb Cortex*. 2012;22(11):2662-2671. doi:10.1093/cercor/bhr344

32. Wilde EA, McCauley SR, Chu Z, et al. Diffusion tensor imaging of hemispheric asymmetries in the developing brain. *J Clin Exp Neuropsychol*. 2009;31(2):205-218. doi:10.1080/13803390802098118

33. Cui Z, Zhong S, Xu P, He Y, Gong G. PANDA: a pipeline toolbox for analyzing brain diffusion images. *Front Hum Neurosci*. 2013;7:42. doi:10.3389/fnhum.2013.00042

34. Rossi S, Hallett M, Rossini PM, Pascual-Leone A, Safety of TMS Consensus Group. Safety, ethical considerations, and application guidelines for the use of transcranial magnetic stimulation in clinical practice and research. *Clin Neurophysiol*. 2009;120(12):2008-2039. doi:10.1016/j.clinph.2009.08.016

5 Author Contributions

TW and YS conceived and designed the study; WD, CL, XY, YZ and LF performed the study and collect materials; HD, CG and YQ analyzed the results; WD, XY and MD wrote the manuscript; YS, TW and WD helped coordinate the study and reviewed the manuscript. All authors contributed to the article and approved the submitted version.

6 Funding statement

This study was funded by the National Key Research &Development Program of China (2022YFC2009700), the Nanjing Municipal Science and Technology Bureau（2019060002) and the National Natural Science Fund (82272612).

7 Patient and Public Involvement

Not applicable. The patient and public will not be involved in the design, conduct, reporting or dissemination of the study.

For peer review only

8 Acknowledgments

We thank all the patients for their contributions during the completion of this study.

Figure 1. Experiment flowchart.

FMA-UE, Fugl-Meyer Assessment of Upper Extremity; AMI, arm sub-score of the Motricity Index; FTHUE-HK, Hong Kong edition of Functional Test for the Hemiplegic Upper Extremity; MBI, Modified Barthel Index; MAS, Modified Ashworth Scale; FA, fractional anisotropy; FAAI, FA asymmetry index; rTMS, repetitive transcranial magnetic stimulation.

Figure 2. Schedule of participant enrollment, interventions, and assessments.

LF-rTMS, low-frequency repetitive transcranial magnetic stimulation; HF-rTMS, high-frequency repetitive transcranial magnetic stimulation; FMA-UE, Fugl-Meyer Assessment of Upper Extremity; AMI, arm sub-score of the Motricity Index; FTHUE-HK, Hong Kong edition of Functional Test for the Hemiplegic Upper Extremity; MBI, Modified Barthel Index; MAS, Modified Ashworth Scale; fMRI, functional magnetic resonance imaging.

For peer review only

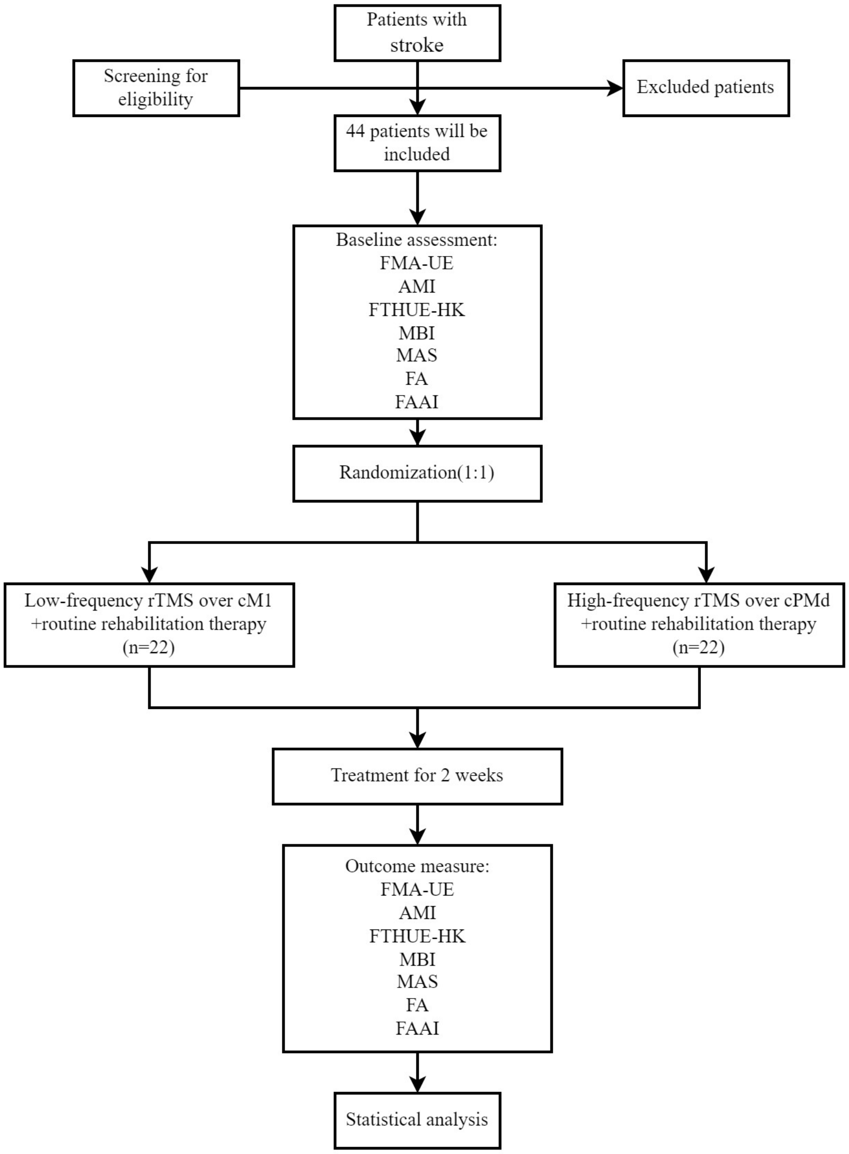

Figure1. Experiment flowchart

460x615mm (72 x 72 DPI)

	Study Period			
	Enrollment	Allocation	Post-allocation	
**TIME POINT**	W-1	0	W1	W2
**ENROLLMENT:**				
Eligibility screen	×			
Informed consent	×			
Randomization	×			
Allocation		×		
**INTERVENTIONS:**				
LF-rTMS protocol over cM1			⟷	⟷
HF-rTMS protocol over cPMd			⟷	⟷
**ASSESSMENTS:**				
FMA-UE	×			×
AMI	×			×
FTHUE-HK	×			×
MBI	×			×
MAS	×			×
fMRI	×			×

Figure2.Schedule of participant enrollment, interventions, and assessments

337x259mm (144 x 144 DPI)

SPIRIT
STANDARD PROTOCOL ITEMS: RECOMMENDATIONS FOR INTERVENTIONAL TRIALS

SPIRIT 2013 Checklist: Recommended items to address in a clinical trial protocol and related documents*

Section/item	Item No	Description
**Administrative information**		
Title	1	Descriptive title identifying the study design, population, interventions, and, if applicable, trial acronym ☑ line1-4, page1
Trial registration	2a	Trial identifier and registry name. If not yet registered, name of intended registry ☑ line38, page1
	2b	All items from the World Health Organization Trial Registration Data Set ☑ line415-419, page11
Protocol version	3	Date and version identifier ☑ line282, page7
Funding	4	Sources and types of financial, material, and other support ☑ line405-407, page11
Roles and responsibilities	5a	Names, affiliations, and roles of protocol contributors ☑ line5-12, page1;
	5b	Name and contact information for the trial sponsor ☑ line13-17, page1
	5c	Role of study sponsor and funders, if any, in study design; collection, management, analysis, and interpretation of data; writing of the report; and the decision to submit the report for publication, including whether they will have ultimate authority over any of these activities ☑ line400-404, page10-11
	5d	Composition, roles, and responsibilities of the coordinating centre, steering committee, endpoint adjudication committee, data management team, and other individuals or groups overseeing the trial, if applicable (see Item 21a for data monitoring committee) ☑ line400-404, page10-11
**Introduction**		
Background and rationale	6a	Description of research question and justification for undertaking the trial, including summary of relevant studies (published and unpublished) examining benefits and harms for each intervention ☑ line50-91, page2-3
	6b	Explanation for choice of comparators ☑ line92-100, page3
Objectives	7	Specific objectives or hypotheses ☑ line100-104, page3

For peer review only

Trial design	8	Description of trial design including type of trial (eg, parallel group, crossover, factorial, single group), allocation ratio, and framework (eg, superiority, equivalence, noninferiority, exploratory) ☑ line106, page3

Methods: Participants, interventions, and outcomes

Study setting	9	Description of study settings (eg, community clinic, academic hospital) and list of countries where data will be collected. Reference to where list of study sites can be obtained ☑ line106-108, page3
Eligibility criteria	10	Inclusion and exclusion criteria for participants. If applicable, eligibility criteria for study centres and individuals who will perform the interventions (eg, surgeons, psychotherapists) ☑ line120-137, page3-4
Interventions	11a	Interventions for each group with sufficient detail to allow replication, including how and when they will be administered ☑ line152-171, page4-5
	11b	Criteria for discontinuing or modifying allocated interventions for a given trial participant (eg, drug dose change in response to harms, participant request, or improving/worsening disease) ☑ line264-271, page7
	11c	Strategies to improve adherence to intervention protocols, and any procedures for monitoring adherence (eg, drug tablet return, laboratory tests) ☑ line288-290, page7
	11d	Relevant concomitant care and interventions that are permitted or prohibited during the trial ☑ line153-157, page4
Outcomes	12	Primary, secondary, and other outcomes, including the specific measurement variable (eg, systolic blood pressure), analysis metric (eg, change from baseline, final value, time to event), method of aggregation (eg, median, proportion), and time point for each outcome. Explanation of the clinical relevance of chosen efficacy and harm outcomes is strongly recommended ☑ line172-212 ,page5
Participant timeline	13	Time schedule of enrolment, interventions (including any run-ins and washouts), assessments, and visits for participants. A schematic diagram is highly recommended (see Figure) ☑ Figure 2 (line419-423, page11)
Sample size	14	Estimated number of participants needed to achieve study objectives and how it was determined, including clinical and statistical assumptions supporting any sample size calculations ☑ line138-144 ,page4
Recruitment	15	Strategies for achieving adequate participant enrolment to reach target sample size ☑ line106-110 ,page3

Methods: Assignment of interventions (for controlled trials)

Allocation:

Sequence generation	16a	Method of generating the allocation sequence (eg, computer-generated random numbers), and list of any factors for stratification. To reduce predictability of a random sequence, details of any planned restriction (eg, blocking) should be provided in a separate document that is unavailable to those who enrol participants or assign interventions ☑ line146-149, page4
Allocation concealment mechanism	16b	Mechanism of implementing the allocation sequence (eg, central telephone; sequentially numbered, opaque, sealed envelopes), describing any steps to conceal the sequence until interventions are assigned ☑ line146-148, page4
Implementation	16c	Who will generate the allocation sequence, who will enrol participants, and who will assign participants to interventions ☑ line149-151, page4
Blinding (masking)	17a	Who will be blinded after assignment to interventions (eg, trial participants, care providers, outcome assessors, data analysts), and how ☑ line149-151, page4
	17b	If blinded, circumstances under which unblinding is permissible, and procedure for revealing a participant's allocated intervention during the trial ☒ only evaluator is blinded

Methods: Data collection, management, and analysis

Data collection methods	18a	Plans for assessment and collection of outcome, baseline, and other trial data, including any related processes to promote data quality (eg, duplicate measurements, training of assessors) and a description of study instruments (eg, questionnaires, laboratory tests) along with their reliability and validity, if known. Reference to where data collection forms can be found, if not in the protocol ☑ line247-249, page6
	18b	Plans to promote participant retention and complete follow-up, including list of any outcome data to be collected for participants who discontinue or deviate from intervention protocols ☑ line248-250, page6
Data management	19	Plans for data entry, coding, security, and storage, including any related processes to promote data quality (eg, double data entry; range checks for data values). Reference to where details of data management procedures can be found, if not in the protocol ☑ line250-252, page6
Statistical methods	20a	Statistical methods for analysing primary and secondary outcomes. Reference to where other details of the statistical analysis plan can be found, if not in the protocol ☑ line213-246, page6

For peer review only - http://bmjopen.bmj.com/site/about/guidelines.xhtml

	20b	Methods for any additional analyses (eg, subgroup and adjusted analyses) ☒ Methods for any additional analyses were not employed in this study.
	20c	Definition of analysis population relating to protocol non-adherence (eg, as randomised analysis), and any statistical methods to handle missing data (eg, multiple imputation) ☑ line225-230, page6

Methods: Monitoring

Data monitoring	21a	Composition of data monitoring committee (DMC); summary of its role and reporting structure; statement of whether it is independent from the sponsor and competing interests; and reference to where further details about its charter can be found, if not in the protocol. Alternatively, an explanation of why a DMC is not needed ☑ line253-262, page7
	21b	Description of any interim analyses and stopping guidelines, including who will have access to these interim results and make the final decision to terminate the trial ☑ line268-271, page7
Harms	22	Plans for collecting, assessing, reporting, and managing solicited and spontaneously reported adverse events and other unintended effects of trial interventions or trial conduct ☑ line263-273, page7
Auditing	23	Frequency and procedures for auditing trial conduct, if any, and whether the process will be independent from investigators and the sponsor ☑ line271-273, page7

Ethics and dissemination

Research ethics approval	24	Plans for seeking research ethics committee/institutional review board (REC/IRB) approval ☑ line275-277, page7
Protocol amendments	25	Plans for communicating important protocol modifications (eg, changes to eligibility criteria, outcomes, analyses) to relevant parties (eg, investigators, REC/IRBs, trial participants, trial registries, journals, regulators) ☑ line278- 282, page7
Consent or assent	26a	Who will obtain informed consent or assent from potential trial participants or authorised surrogates, and how (see Item 32) ☑ line283-290, page7
	26b	Additional consent provisions for collection and use of participant data and biological specimens in ancillary studies, if applicable ☒This content wasn't involved in the study.
Confidentiality	27	How personal information about potential and enrolled participants will be collected, shared, and maintained in order to protect confidentiality before, during, and after the trial ☑ line250-252, page6
Declaration of interests	28	Financial and other competing interests for principal investigators for the overall trial and each study site ☑ line291-293, page7-8

review only

Access to data	29	Statement of who will have access to the final trial dataset, and disclosure of contractual agreements that limit such access for investigators ☑ line294-297, page8
Ancillary and post-trial care	30	Provisions, if any, for ancillary and post-trial care, and for compensation to those who suffer harm from trial participation ☒ N/A
Dissemination policy	31a	Plans for investigators and sponsor to communicate trial results to participants, healthcare professionals, the public, and other relevant groups (eg, via publication, reporting in results databases, or other data sharing arrangements), including any publication restrictions ☑ line298-300, page8
	31b	Authorship eligibility guidelines and any intended use of professional writers ☑ line298-300, page8
	31c	Plans, if any, for granting public access to the full protocol, participant-level dataset, and statistical code ☒ There weren't plans for granting public access to the full protocol, participant-level dataset, and statistical code.
**Appendices**		
Informed consent materials	32	Model consent form and other related documentation given to participants and authorised surrogates ☑ the patient consent form in the supplementary file
Biological specimens	33	Plans for collection, laboratory evaluation, and storage of biological specimens for genetic or molecular analysis in the current trial and for future use in ancillary studies, if applicable ☒ There weren't plans for collection, laboratory evaluation, or storage of biological specimens in this study.

*It is strongly recommended that this checklist be read in conjunction with the SPIRIT 2013 Explanation & Elaboration for important clarification on the items. Amendments to the protocol should be tracked and dated. The SPIRIT checklist is copyrighted by the SPIRIT Group under the Creative Commons "Attribution-NonCommercial-NoDerivs 3.0 Unported" license.

For peer review only
